# Beyond protein synthesis: the emerging role of arginine in poultry nutrition and host-microbe interactions

**DOI:** 10.3389/fphys.2023.1326809

**Published:** 2024-01-03

**Authors:** Shahna Fathima, Walid Ghazi Al Hakeem, Ramesh K. Selvaraj, Revathi Shanmugasundaram

**Affiliations:** ^1^ Department of Poultry Science, University of Georgia, Athens, GA, United States; ^2^ United States Department of Agriculture, Athens, GA, United States

**Keywords:** arginine, poultry, gut health, nutraceuticals, immune response

## Abstract

Arginine is a functional amino acid essential for various physiological processes in poultry. The dietary essentiality of arginine in poultry stems from the absence of the enzyme carbamoyl phosphate synthase-I. The specific requirement for arginine in poultry varies based on several factors, such as age, dietary factors, and physiological status. Additionally, arginine absorption and utilization are also influenced by the presence of antagonists. However, dietary interventions can mitigate the effect of these factors affecting arginine utilization. In poultry, arginine is utilized by four enzymes, namely, inducible nitric oxide synthase arginase, arginine decarboxylase and arginine: glycine amidinotransferase (AGAT). The intermediates and products of arginine metabolism by these enzymes mediate the different physiological functions of arginine in poultry. The most studied function of arginine in humans, as well as poultry, is its role in immune response. Arginine exerts immunomodulatory functions primarily through the metabolites nitric oxide (NO), ornithine, citrulline, and polyamines, which take part in inflammation or the resolution of inflammation. These properties of arginine and arginine metabolites potentiate its use as a nutraceutical to prevent the incidence of enteric diseases in poultry. Furthermore, arginine is utilized by the poultry gut microbiota, the metabolites of which might have important implications for gut microbial composition, immune regulation, metabolism, and overall host health. This comprehensive review provides insights into the multifaceted roles of arginine and arginine metabolites in poultry nutrition and wellbeing, with particular emphasis on the potential of arginine in immune regulation and microbial homeostasis in poultry.

## 1 Introduction

Amino acids are organic compounds containing both amino (—NH_2_) and carboxyl (—COOH) groups. Due to the presence of asymmetric carbon, all amino acids except glycine exhibit optical activity and exist as D- and L-isoforms or enantiomers (Lehninger et al., 2005). The asymmetric α-carbon imparts chirality, a phenomenon where the molecule is not superimposable to its mirror images in space. Due to this, amino acids except glycine exist in different stereoisomeric forms (Grishin et al., 2020). The amino acids’ biochemical properties and physiological functions vary widely depending on the side chains, which impart charge to the amino acids and their isoforms (Wu, 2009; Grishin et al., 2020). There are 20 amino acids that function as building blocks of proteins in animal tissues. Based on their dietary requirements, amino acids are broadly classified as essential (indispensable) and non-essential (dispensable) for the growth and nitrogen balance of the animal. Essential amino acids are derived from the diet, as the organism cannot synthesize the carbon skeleton of those amino acids or synthesize them in amounts not adequate to meet the requirements (Watford, 2008; Wu, 2009). Conversely, non-essential amino acids can be synthesized *de novo* by the organism in sufficient amounts to meet the requirements in a species-dependent manner (Lehninger et al., 2005; D'mello, 2003a). However, some amino acids that are traditionally considered non-essential, are required in increased amounts under some pathological conditions, necessitating dietary supplementation, and are termed conditionally essential amino acids (D'mello, 2003b).

Recently, the concept of functional amino acids was introduced by Wu, (2009). Functional amino acids can be nutritionally essential, non-essential, or conditionally essential during different physiological stages of the animal. Functional amino acids play a pivotal role in gene expression (Leong et al., 2006), oxidative homeostasis, and cell signaling (Wang et al., 2008) and they regulate various physiologic and metabolic processes such as growth, development, immunity, health, reproduction, and endocrine status (Leong et al., 2006; Wang et al., 2008; Tan et al., 2009; Jimoh et al., 2021). Functional amino acids essential to maintaining whole-body homeostasis include methionine, arginine, proline, glutamine, leucine, glycine, tryptophan, and cysteine (Wu, 2010). These amino acids exert their functional roles directly or through their metabolites, exerting antioxidant, immunomodulatory, and growth-promoting effects (Fagundes et al., 2020; Liu et al., 2023). The cellular mechanisms by which these amino acids, notably arginine (Rubin et al., 2007; Tan et al., 2009; Tan et al., 2010; Al-Daraji and Salih, 2012; Tan et al., 2014; Zhang et al., 2017; Dao et al., 2022a), exert their beneficial effects, their functional roles, and their potential use as nutraceuticals in poultry feed have been investigated lately.

Arginine is a functional amino acid essential for growth, energy metabolism, immune response, wound healing, and protein synthesis (Wu et al., 2009). Additionally, arginine is the precursor for various bioactive molecules such as NO, polyamines, agmatine, creatine, glutamine, glutamate, and proline (Almquist et al., 1941; Montanez et al., 2008). Supplementation of arginine and its metabolites such as guanidinoacetic acid (GAA) and citrulline in poultry feed improves growth performance, carcass yield, lean meat yield, bone development, immunity, and antioxidant capacity (Al-Daraji and Salih, 2012; Tan et al., 2014; Chowdhury et al., 2017; Zhang et al., 2018; Dao et al., 2021a; Dao et al., 2022a). This review article delves into the intricate facets of arginine, shedding light on its absorption, metabolism, and physiologic functions in poultry. This review also briefly explores the commercially available arginine metabolites, GAA, and citrulline, shedding light on their roles within the broader context of poultry physiology and health. A particular emphasis is given to the interaction of arginine with the gut microbial community during health and disease, with a specific focus on necrotic enteritis as the disease model. Therefore, this review aims to offer an encompassing perspective on the present understanding of arginine’s functional role in enhancing poultry health and production.

## 2 Arginine in poultry production

**FIGURE 1:**
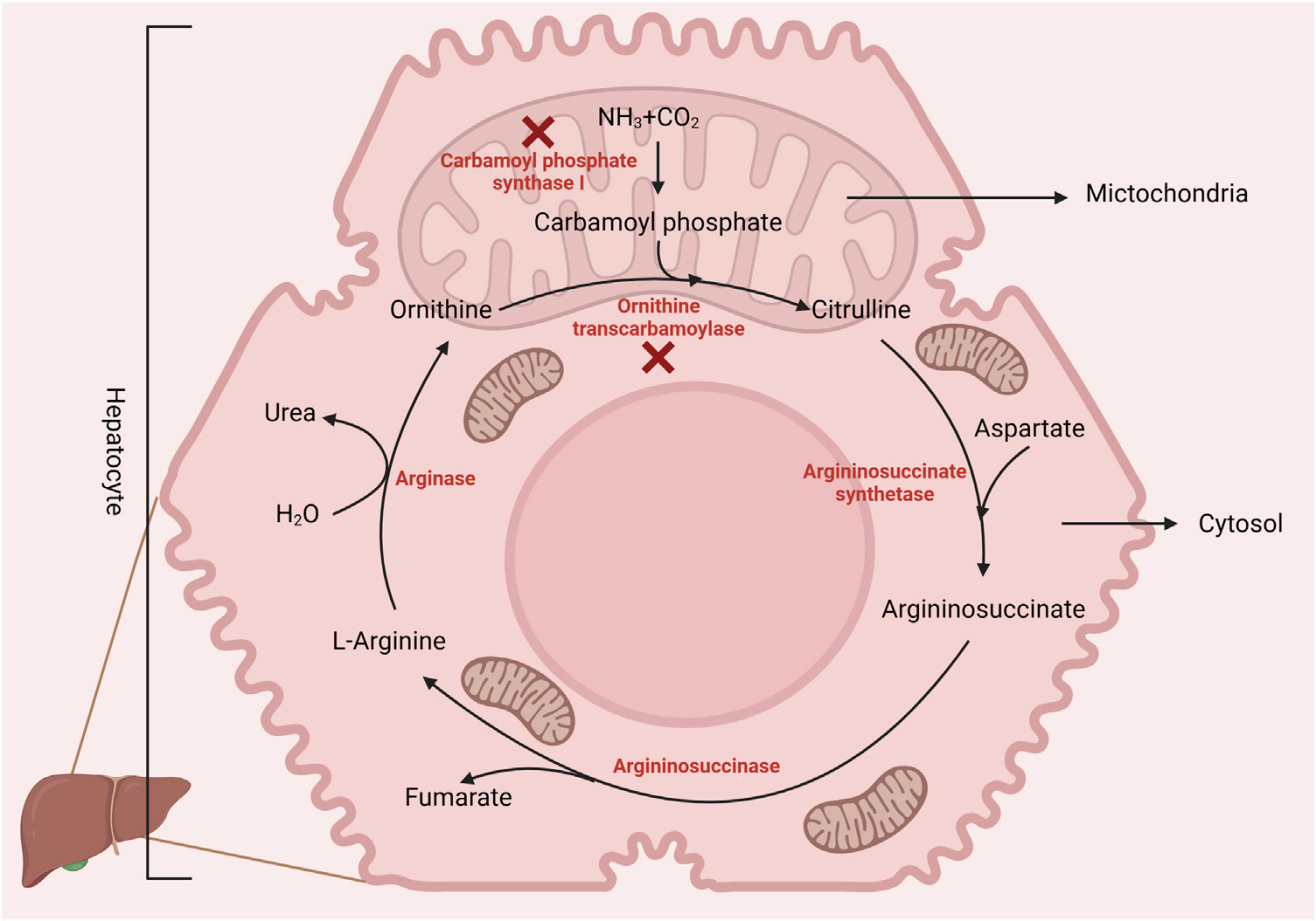
Birds lack the enzyme carbamoyl phosphate synthase-1 (incomplete urea cycle), making L-arginine dietary essential. However, poultry can synthesize arginine from citrulline via arginosuccinate synthase and lyase. Created with biorender.com (21 May 2022).

Arginine is a dibasic amino acid (Rubin et al., 2007) consisting of a linear chain of four carbon molecules and a distal complex guanidinium group, displaying resonance hybrid properties that impart the chemical properties of arginine (Khajali and Wideman, 2010). Arginine is an essential amino acid in poultry due to the absence of a functional urea cycle (as illustrated in Figure 1) (Application of Nutritional Immunology, 2022). This dietary indispensability of arginine in chickens arises from the lack of the enzyme carbamoyl phosphate synthase-I, which is necessary for the synthesis of L-arginine from ornithine, ammonia, and amino-nitrogen of aspartate. Additionally, poultry exhibits lower activities of ornithine transcarbamoylase and hepatic arginase (Khajali and Wideman, 2010), reinforcing their dependency on dietary arginine. Nevertheless, in the presence of dietary citrulline, arginine synthesis can occur in chicken macrophages and kidneys (Allen and Fetterer, 2000). Citrulline can replace arginine in the diet because of argininosuccinate and argininosuccinate synthetase enzymes in poultry. However, due to the lack of the enzyme carbamoyl phosphate synthetase, chicks cannot utilize ornithine (Tamir and Ratner, 1963a) as a source of arginine. In addition, hepatic arginine synthesis does not occur in chickens as the arginase activity is relatively higher in the liver.

Arginine is the fifth-limiting amino acid in a corn-soybean meal-based poultry diet. The National Research Council (NRC) requirement of arginine for broilers is 1.25%, 1.10%, and 1.00% of the diet for up to 3 weeks, 3–6 weeks, and 6–8 weeks of age, respectively (National Research Council, 1994b). However, the last updated version of the NRC recommendations for poultry was published in 1994. Commercial broilers were genetically selected in the last few decades to improve body weight gain, feed efficiency, and breast muscle yield (Applegate and Angel, 2014). The requirements for this increased growth and production performance, changes in management practices, and feed-related changes have not been accounted for in the NRC (1994) recommendations (Applegate and Angel, 2014). According to recent research findings, the NRC recommendations for arginine might not be adequate to support increased growth, prevent pulmonary hypertension due to stressful environmental conditions, and support arginine-dependent immune responses (Khajali and Wideman, 2010). However, other studies suggest the arginine requirements are close to the NRC (1994) recommendations (Jahanian, 2009). Nonetheless, the arginine requirement for optimum cellular and humoral immune responses in poultry is thought to be higher than that required for maximum growth rate in poultry (Jahanian, 2009).

Arginine is not a limiting amino acid in a corn-soybean meal-based poultry diet with an arginine: lysine ratio ranging from 100 to 107 (2022-Cobb500-Broiler-Performance-Nutrition-Supplement, 2022; Ross-BroilerNutritionSpecifications2022-EN, 2022). However, recent studies indicate that a higher arginine: lysine ratio is recommended for improved BWG and FCR (Zampiga et al., 2018; Sirathonpong et al., 2019; Corzo et al., 2021). Arginine supplementation is also recommended when birds are raised at high altitudes, during heat and cold stress, and when increasing the stocking density (Brake et al., 1998; SRINONGKOTE et al., 2004; Kodambashi Emami et al., 2017). The increased use of low crude protein feed formulations, replacement of soybean meal with by-products such as corn distillers’ dried grains, and reduced use of animal sources of protein in poultry diet necessitates arginine supplementation (DeGroot et al., 2018). The requirement for arginine can vary depending on several other factors, such as dietary protein level, source of protein, digestibility of feed ingredients, stage of growth, and physiological status of the bird (McNab, 1994).

The proteins’ nutritional value and amino acid composition vary with the dietary ingredients used in poultry feed formulation. Amino acid availability is a valuable measure and indicator of protein quality (McNab, 1994; Ravindran et al., 1999) Estimating the amino acid availability of feed ingredients enables the efficient formulation of poultry feed, accounting for endogenous losses. Amino acid availability is defined as “the proportion of dietary amino acids that is in a form suitable for digestion, absorption, and utilization by the animal” (McNab, 1994). Amino acid digestibility is a sensitive indicator of dietary amino acid availability for poultry. Excreta analysis is the most common method used to assess the amino acid digestibility in poultry. Nonetheless, since the urine and feces are excreted together in poultry, excreta analysis measures amino acid metabolizability rather than digestibility (Ravindran et al., 1999). Analysis of ileal contents is a more reliable method for assessing amino acid digestibility in poultry as it takes into account the hindgut fermentation, preventing underestimation of the amino acid requirement (Investigation of protein quality, 1968). In addition, there are differences in the amino acid digestibility among different feed ingredients. Amino acid digestibility is highest in oilseed meals, particularly soybean and sunflower meals. Arginine digestibility was highest in oilseed meals (except for cottonseed meal), grain legumes, wheat middling, and rice polishings. Among animal protein sources, blood meal had the highest amino acid digestibility coefficient, followed by fish meal, meat meal, meat, bone meal, and feather meal, respectively (Ravindran et al., 2005). These factors affecting the digestibility and estimation of the digestibility of arginine influence the arginine requirement of the birds as well.

In growing chicks, the requirement for arginine will be greatly increased (when expressed as g/day) with high demand for the amino acid for muscle protein accretion (Ball et al., 2007). Similarly, during infections, the immune system is activated, which significantly affects the amino acid availability for muscle protein accretion, thus compromising growth. Arginine requirement will be increased during an active inflammatory response (Rochell et al., 2017; Nogueira et al., 2021) indicated by a decreased plasma arginine concentration in infected birds. The increased demand for arginine during enteric infections such as coccidiosis might be due to its role in polyamine synthesis which is required for mucosal tissue repair (Rochell et al., 2017). Thus, the body prioritizes the immune response over protein deposition during stress conditions (Nogueira et al., 2021) reducing the metabolic availability of arginine and negatively impacting growth (Allen and Fetterer, 2000). Similarly, during stress conditions, especially during heat stress, the sodium-dependent and sodium-independent uptake of arginine is depressed, increasing the arginine requirement in birds (Khajali and Wideman, 2010). The requirement for arginine can also vary based on its relationship other amino acids such as lysine and methionine (Chamruspollert et al., 2002), methodology used to estimate the requirement, and type of birds used (Lima et al., 2020). However, determining the actual requirement for arginine hinges on its bioavailability, contingent upon both digestibility and post-digestion utilization of the amino acid. Thus, comprehending the intricate processes of absorption, transport, and metabolism of arginine in poultry is crucial, and these aspects will be explored further in the subsequent sections.

## 3 Arginine absorption and transport mechanisms

Arginine, being a cationic amino acid, shares transport proteins with other cationic amino acids such as ornithine and lysine (Closs and Mann, 2000). The carrier proteins for cationic amino acids belong to the solute carrier family 7 (SLC7) and include members 1,7 and 9. The sodium-independent transporter SLC7A1 preferably transports arginine, followed by lysine and histidine. However, the sodium-dependent transporters SLC7A7 and SLC7A9 prefer lysine (Bröer and Fairweather, 2018). Arginine, in most cells, is taken up by a Na^+^-independent transport system, termed system y^+^. The system y^+^ is constituted by the cationic amino acid transporter (CAT) proteins CAT-1, CAT-2B, and CAT-3 (Closs et al., 2004). The CAT transporters cater to the cationic amino acid requirements for protein synthesis and the synthesis of bioactive substances such as NO, creatine, proline, polyamines, agmatine, glutamine, and urea (San Martín and Sobrevia, 2006). The glycoprotein-associated heterodimeric b^0,+^AT/rBAT transporter is a Na^+^-independent transporter located in the luminal side of the epithelium and facilitates the inward transport of dibasic amino acids such as arginine and lysine in exchange for neutral amino acids (Torras-Llort et al., 2001). The neutral amino acids necessary for exchange with arginine are transported by PepT1, PepT2, or B^0^AT and y^+^LAT1 at the apical and basolateral membranes, respectively (Closs et al., 2004). In addition, amino acid transporters are present on the basolateral membrane of the enterocytes that facilitate the exchange of amino acids between the vascular system and the epithelial cells. The transporters y^+^ LAT1 and y^+^ LAT2 transport neutral and cationic amino acids, whereas CAT1 and CAT2 transport cationic amino acids across the basolateral membrane of the enterocytes (Miska and Fetterer, 2017).

The expression of these amino acid transporters is significantly decreased during infections, leading to malabsorption, weight loss, and immune dysfunction (Miska and Fetterer, 2017). Further, intestinal immunopathology is significantly increased during infection-associated arginine deficiency. This infection-induced damage can be reversed by administering supplemental arginine (Zhang et al., 2019). During such conditions, arginine is mobilized from body protein to satisfy the increased demand or to compensate for the decreased availability (Faure et al., 2007). However, the absorption and utilization of arginine is also influenced by the amino acid balance, acid-base balance, and the presence of antagonists in the diet (Jones et al., 1967; Khajali and Wideman, 2010). These interrelationships of arginine with other dietary components affecting its absorption and utilization are discussed below.

## 4 Nutritional antagonism: interaction of other amino acids with arginine

A dietary balance of micronutrients, such as essential amino acids, is important for optimum growth and development (Zampiga et al., 2018). The amino acids interact with each other to maximize the growth and production performance in poultry (Kidd et al., 1997). A change in the dietary inclusion level of one amino acid can cause a marginal deficiency of other amino acids if the balance is not maintained. Antagonism occurs due to the competition among amino acids for absorption and transport systems and common enzymes used in their catabolism due to similarities in their chemical structures. Moreover, antagonists might inhibit the uptake or utilization of the amino acid, affecting its availability for physiological functions. Factors such as dietary imbalances in amino acid composition, competition for transporters, or metabolic interactions can contribute to amino acid antagonism (Bell, 2003; Maynard and Kidd, 2022).

The balance between arginine and lysine is important in poultry feed. The nutritional antagonism of arginine and lysine was first identified in 1952 (Anderson and Combs, 1952). The antagonism is explained by the fact that arginine and lysine are basic amino acids competing for renal tubular reabsorption. The antagonism is more pronounced with excess lysine than with excess arginine. The antagonistic effects are observable when the lysine content in the poultry diet is approximately 2%–3.5% or when the lysine-to-arginine ratio is 2.2–2.6: 1 (Ball et al., 2007). A high lysine: arginine ratio enhances renal arginase activity, leading to increased degradation and urinary excretion of arginine (Khajali and Wideman, 2010). Excess lysine affects the muscle amino acid concentration and growth in poultry. The suppression of weight gain by diets high in lysine was first reported by Anderson and Combs (Anderson and Combs, 1952) whereas, the growth-depressing effect of a high arginine diet was first reported by D’mello and Lewis (D’mello and Lewis, 1970). The optimum dietary arginine: lysine ratio recommended by the NRC is 1.14, 1.10, and 1.18 for 0–3 weeks, 3–6 weeks, and 6–8 weeks respectively (National Research Council, 1994a). The effect of high lysine on arginine can be attenuated by supplementing sodium, potassium, calcium, or magnesium salts of organic acids, such as sodium and potassium acetate (Khajali and Wideman, 2010).

In contrast to the above-discussed findings, in a study conducted by Kadirvel and Kratzer (1974), it was observed that leucine significantly inhibited the uptake of arginine more than lysine. This antagonism can be due to the faster absorption of leucine and lysine. Arginine deficiency produced by lysine can be due to the metabolic effect rather than competitive inhibition of intestinal absorption as well (Jones et al., 1967). Kidney arginase activity and urea excretion have a significant impact on arginine requirements and homeostasis. Several amino acids such as lysine, isoleucine, phenylalanine, histidine, and tyrosine significantly increase kidney arginase activity (Austic and Nesheim, 1970) while glycine and threonine suppress kidney arginase activity (Austic and Nesheim, 1970).

**FIGURE 2:**
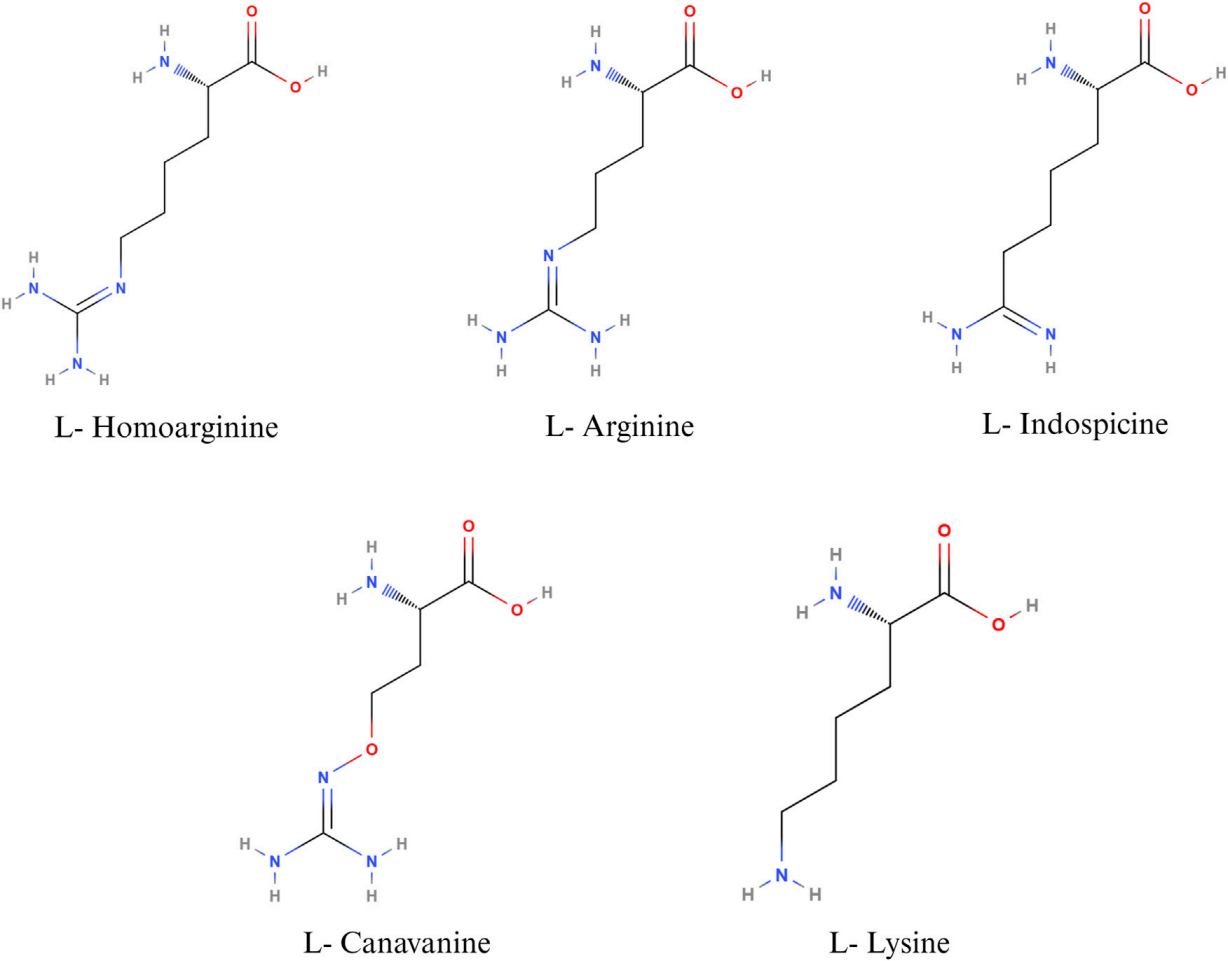
Nutritional antagonists of arginine. Created with BioRender.com (26 November 2023).

Non-protein amino acids such as canavanine, homoarginine, and indospicine are structural analogues of arginine (Figure 2), implicated in antagonistic activity against arginine. Canavanine is a non-protein structural analogue of arginine and is found predominantly in legumes and crops such as alfalfa, clover, bitter vetch, and trefoils. The seed of bitter vetch contains 28.5% crude protein and can be used as an alternative source of protein in poultry feeds. However, the presence of canavanine limits its use as an alternative feedstuff in monogastric animals (Sadeghi et al., 2004; Sadeghi et al., 2009a; Sadeghi et al., 2009b). Canavanine is stored in leguminous plants as a chemical barrier against diseases causing pathogens and predation. In animals, canavanine can replace arginine during protein synthesis, leading to the synthesis of non-functional proteins (Sadeghi et al., 2009b). In addition, canavanine replaces ornithine in the urea cycle, leading to the formation of canavaninosuccinate. Canavaninosuccinate inhibits the ornithine decarboxylate enzyme, hindering the biosynthesis of polyamines such as spermine, spermidine, and putrescine (D'mello, 2003a). Canavanine also inhibits Na^+^- dependent transport of arginine across the intestinal epithelium (Khajali and Wideman, 2010). Canavanine in poultry feed can adversely affect growth performance and cause pancreatic hypertrophy (Sadeghi et al., 2004). However, canavanine in the feedstuffs can be inactivated by different treatment methods, primarily soaking, acid treatment, alkali treatment, or heat treatment (Sadeghi et al., 2004).

L-homoarginine, a non-protein amino acid, is synthesized from the catabolism of lysine or transamination of arginine in the small intestine, liver, and kidneys (Adams et al., 2019). Homoarginine can affect NO production by acting as a substrate for the enzyme NOS. As L-Homoarginine uses the same intestinal amino acid transporter as lysine, feeding homoarginine was found to decrease feed consumption in birds and cause lysine deficiency in rats (Adams et al., 2019). Homoarginine acts as a competing substrate for the enzymes that use arginine as a substrate (Haghikia et al., 2017). The effect of homoarginine on nitric oxide production can be positive or negative, depending on several factors, such as the cell type, intracellular and extracellular concentrations of arginine, and the activity of other arginine metabolizing enzymes. Feeding homoarginine in poultry inhibits the secretion of alkaline phosphatase, which is important for the maintenance of gut health and intestinal homeostasis (Adams et al., 2019). Alkaline phosphatase plays an important role in bone formation as well; hence, homoarginine levels are inversely proportional to the parameters of bone formation (Linder, 2016; Adams et al., 2019). Hence, homoarginine plays an important role in different metabolic processes in poultry. However, the normal level of serum homoarginine and its implications for poultry health and wellbeing have not been well established.

Indospicine, a non-proteinogenic amino acid, is a competitive inhibitor of arginine. Indospicine is found in *Indigofera* plant species. The compound causing *Indigofera* toxicity was identified in 1970 as indospicine by Hegarty and Pound (Hegarty and Pound, 1970). Indospicine acts as a cation on ion exchange resins, is not metabolized by arginase, and interferes with the incorporation of arginine in proteins (Hegarty and Pound, 1970; Bell, 2003). Livestock, grazing on pasture, accumulates toxins in their meat, which leads to the secondary poisoning of animals consuming the meat of grazers. Indospicine interferes with the arginine metabolic pathways in mammals and is highly hepatotoxic and teratogenic (Fletcher et al., 2015). In poultry, feeding 5% *I. spicata* meal caused decreased growth rate and paralysis of the neck, wings, and legs, followed by death (Rosenberg and Zoebisch, 1952). It has been suggested that poultry, being a uricotelic species, is less susceptible to the adverse effects of indospicine than ureotelic animals (Bell, 2003). Nevertheless, the current literature on the effect of indospicine on poultry health is sparse.

## 5 Arginine metabolism and physiological effects of metabolites in poultry

**FIGURE 3:**
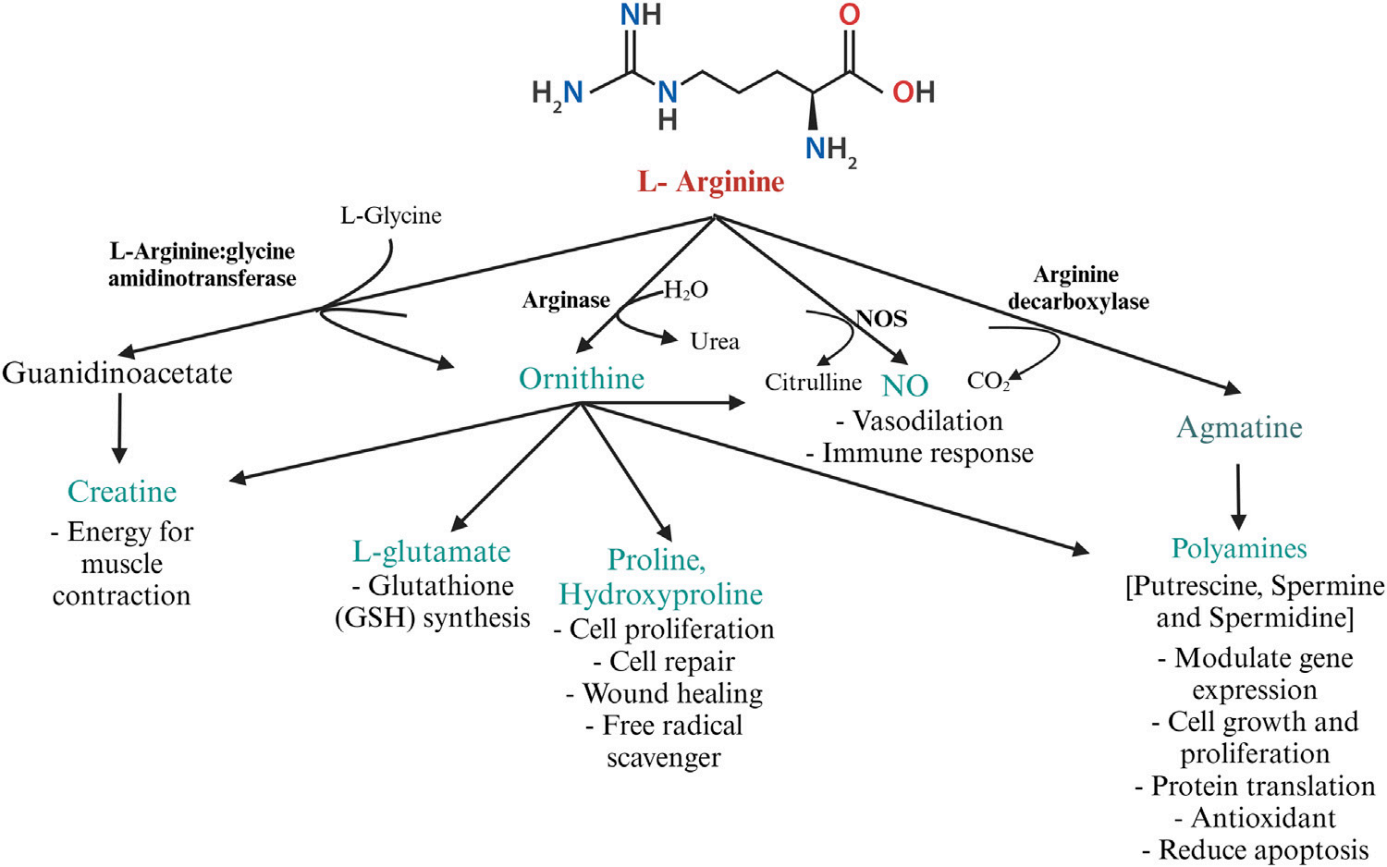
Metabolism of arginine by the major arginine metabolizing enzymes L-arginine: glycine amidinotransferase, arginase, NOS, and arginine decarboxylase in poultry. Created with Biorender.com (8 October 2022).

In poultry, the fate of arginine is determined by the activities of CATs, arginosuccinate synthase, and the arginine-degrading enzymes- NOS and arginases (Wu and Morris, 1998). The key enzymes involved in arginine catabolism are 1. NOS, 2. arginase 3. arginine decarboxylase (ADC), and 4. arginine: glycine amidinotransferase (AGAT), summarized in Figure 3. The expression of these enzymes is cell-specific. The three isozymes of NOS, namely, neuronal NOS (nNOS or NOS 1), endothelial NOS (eNOS or NOS 3), and iNOS or NOS2 differ in their structure, distribution, and synthetic capacity, but catalyze the same reaction (Stuehr et al., 2004). The enzyme NOS incorporates molecular oxygen at the terminal guanidino nitrogen group of arginine, yielding NO and citrulline. The gene expressions of nNOS and eNOS are constitutive, whereas the expression of iNOS is inducible. While nNOS and eNOS-mediated production of NO is “low-output” and is important for normal physiological functions, the production of NO by iNOS is classified as “high-output” and is involved in infection and inflammation (MacMicking et al., 1997).

Under physiological conditions, NO (produced by the expression of eNOS and nNOS) is necessary for vasodilation, parasympathetic neuronal action, smooth muscle relaxation, spermatogenesis, gene expression, and embryogenesis in poultry. For instance, *in ovo*, inoculation of arginine in chicks improves egg weight, hatchability, chick weight, production performance, lymphoid organ weight, and liver and pectoral muscle energy storage (Nabi et al., 2022), that might contribute to the increased survivability of chicks. In addition, arginine supplementation in poultry raised at high altitudes helps to regulate vasodilation and prevent heart disease and subsequent ascites syndrome in poultry (Miri et al., 2022) due to the production of NO. eNOS expression is upregulated during hypertension, hypoxia, and hypoxemia. This will promote calcium entry into the endothelial cells transiently, which forms the calcium-calmodulin complex and stimulates NO production. NO acts as a vasodilator, relieving hypertension and increasing the blood supply to the tissues (Bowen et al., 2007). However, during inflammation, pro-inflammatory cytokines such as IFN-γ, IL-1β, IL-12, tumor necrosis factor- α (TNF-α), and bacterial lipopolysaccharides (LPS) induce the expression of iNOS (Qureshi, 2003; Bowen et al., 2007). The activity of the different isoforms of NOS has been reviewed previously (MacMicking et al., 1997). Arginine is the only known substrate for all three isoforms of NOS and the precursor of NO in the body (Wu and Morris, 1998) and hence, NOS competes with other arginine-degrading enzymes, such as arginase.

Arginase exists predominantly in two isoforms-liver-type arginase I and non-hepatic-type arginase II. Arginase I is present in the cytosol of hepatocytes and erythrocytes, whereas arginase II is present in the mitochondrial matrix of enterocytes and the cells of the kidney. In poultry, arginase activity is highest in the kidney, liver, and macrophages (Tamir and Ratner, 1963b). Expression of arginase I in macrophages is induced by the cytokines IL-4 and IL-13. Arginase downregulates NO production by competing with NOS for arginine (MacMicking et al., 1997). Prolonged production of NO is toxic to macrophages and other cells in the vicinity. Arginase, induced during the later stages of inflammation, depletes intracellular arginine, thus preventing the overproduction of NO. Arginase acts on L-arginine, yielding ornithine, which is decarboxylated by ornithine decarboxylase to form putrescine (MacMicking et al., 1997). In the presence of decarboxylated S-adenosylmethionine, spermine and spermidine can be formed from putrescine by ornithine decarboxylase and S-adenosylmethionine decarboxylase (Seiler, 1987). The polyamines spermine, spermidine, and putrescine are associated with cell repair, cell proliferation, and wound healing (Wu and Morris, 1998). Ornithine can be converted to pyrroline-5-carboxylate further converted to proline and hydroxyproline. Proline and its metabolites regulate gene expression, mTOR pathway (van Meijl et al., 2010), protein synthesis, cell survival, and scavenge free radicals (Kaul et al., 2008). Besides, hydroxyproline is required for the synthesize of glycine, glucose, and pyruvate and is known to scavenge free radicals (Phang et al., 2008).

Decarboxylation of arginine by the mitochondrial enzyme ADC yields the cationic amine agmatine. Agmatine is a precursor for synthesizing polyamines and is important for maintaining mitochondrial membrane permeability (Akasaka and Fujiwara, 2020). Agmatine is a pleiotropic molecule involved in various physiological functions such as NO synthesis, polyamine metabolism, glucose metabolism, carnitine synthesis, and neurotransmission (Molderings and Haenisch, 2012). Agmatine has been discovered to have therapeutic applications and is considered a nutraceutical in mammals (Molderings and Haenisch, 2012). The role of agmatine in poultry is largely unexplored. ADC activity is highest in the kidney and liver. Studies on agmatine revealed the antagonistic activity of agmatine aldehyde on NOS (Satriano, 2004). Agmatine inhibits polyamine biosynthesis by binding to the enzyme ornithine decarboxylase and promoting its degradation. Additionally, agmatine induces the antizyme- I, an enzyme that converts higher-order polyamines to lower-order polyamines (spermine → spermidine → putrescine) (Satriano, 2004). Thus, in general, agmatine possesses antiproliferative and anti-inflammatory activity.

Creatine cannot be synthesized in birds *de novo*. The creatine balance in poultry is dependent on dietary arginine. The enzyme transamidinase (AGAT) catalyzes the transfer of an amidino group from arginine to the N-terminal amine of glycine to yield ornithine and guanidinoacetate (GAA). Guanidinoacetate methyltransferase catalyzes the methyl group transfer from S-adenosylmethionine to GAA, yielding creatine (Portocarero and Braun, 2021). Creatine plays a pivotal role in energy metabolism by acting as a phosphate reservoir for adenosine triphosphate (ATP) formation (Portocarero and Braun, 2021). Though creatine can be endogenously synthesized in mammals, birds fully rely on dietary sources. Creatine is highly unstable and is not approved as a feed additive for poultry. However, GAA acts as a precursor of creatine and is approved as a feed additive in broilers (Majdeddin et al., 2020).

## 6 Arginine sparing effects of arginine metabolites

The lack of commercially available, economical sources of L-Arginine prompted the use of arginine metabolites that fuel arginine’s non-protein functions, sparing more arginine for muscle protein accretion (DeGroot et al., 2018). Citrulline and GAA are metabolites of arginine that are commercially available and exhibit arginine-sparing effects. GAA, also known as glycocyamine, is formed from arginine and glycine by the activity of the enzyme arginine: glycine amidinotransaminase in the kidneys. GAA is methylated in the liver by the action of the enzyme guanidinoacetate-N-methyltransferase to form creatine (Khajali et al., 2020). Creatine is transported to the tissues with high energy demands such as the skeletal muscles, spermatozoa, brain, heart, and retina. Creatine and phosphocreatine play a significant role in cellular energy metabolism through the formation of high-energy phosphate bonds (Lemme et al., 2007). However, the tissues have a limited storage capacity for creatine and hence, high circulating creatine levels induce a negative feedback mechanism that inhibits the formation of GAA (Khajali et al., 2020). Though creatine synthesis represents a major proportion of arginine utilization, the thermal instability of creatine limits its use as a feed additive (Vraneš et al., 2017; Khajali et al., 2020). Synthetic GAA is highly thermostable and has a high recovery rate from feed, making it a suitable feed additive in pelleted and extruded feed (Vraneš et al., 2017). GAA supplementation also bypasses the negative feedback inhibition by creatine (DeGroot et al., 2018). Hence, GAA can be considered as a readily available source of creatine and can reduce or spare arginine requirement in broilers (Arginine sparing potential of guanidinoacetic acid in broiler nutrition, 2018). DeGroot, A. A., Braun, U., & Dilger, R. N. (2018) demonstrated that supplementation of 0.12% GAA in an arginine-deficient diet fed to broiler chickens reversed the arginine deficiency-induced reduction in growth performance, muscle glycogen concentration, and muscle phosphagen concentration (DeGroot et al., 2018). GAA supplementation in a low-protein diet during heat stress in chickens improves growth performance and feed conversion ratio (Amiri et al., 2019). GAA supplementation also improves sperm concentration and motility and decreases sperm abnormality in broiler breeder roosters, contributing to improved semen quality and fertility. Creatine phosphate is important for the energy homeostasis of sperm and is required for sperm motility (Tapeh et al., 2017). Creatine also has anti-apoptotic and anti-oxidative effects on cells, which aids in maintaining the plasma membrane integrity of spermatozoa and preventing abnormalities (Meyer et al., 2006). Creatine supplementation also plays a significant role in muscle development, indicated by an improved feed: gain ratio in broilers supplemented with creatine monohydrate. GAA supplementation in energy energy-deficient diet partially reverses the adverse effects of dietary energy reduction on the growth performance of poultry (Fosoul et al., 2018). Supplementation of GAA, even in arginine-sufficient diets, is found to have an arginine-sparing effect, diverting arginine from creatine formation to protein accretion in broilers (Portocarero and Braun, 2021). Supplementation of GAA in an adequate protein diet for broilers improves BWG and FCR, which might be due to increased energy efficiency. Moreover, GAA also promotes polyamine synthesis required for the synthesis of RNA, DNA, and proteins, and promotes the production of growth hormones (Ahmadipour et al., 2018).

Citrulline is a non-protein amino acid formed from arginine by the action of the enzyme nitric oxide synthase. Citrulline can be converted to arginine by the sequential action of the enzymes argininosuccinate synthase and argininosuccinate lyase (Chowdhury et al., 2017). Dietary arginine is metabolized by the hepatic arginase during first-pass metabolism or is degraded by the intestinal mucosal arginase, limiting its presence in plasma (Zheng et al., 2017). Citrulline bypasses the hepatic metabolism and can be converted to arginine in the kidneys and released into the bloodstream (El-Hattab et al., 2012). Hence, citrulline can be used for the *de novo* synthesis of arginine in poultry (Uyanga et al., 2023). Several studies in human subjects highlight the therapeutic applications of citrulline in different conditions such as skeletal muscle atrophy (Ham et al., 2015), metabolic syndrome (Sailer et al., 2013), and urea cycle disorders (Johnson, 2017). However, the potential role of citrulline in poultry health and disease is not understood completely. Citrulline can be used to partially replace arginine in broiler diets without causing a detrimental effect on the growth performance and intestinal health of the birds (Uyanga et al., 2023). Citrulline supplementation in poultry increases the activity of the NOS enzyme, improves antioxidant synthesis, reduces lipid peroxidation, and modulates the availability of the free amino acids arginine, ornithine, and citrulline (Uyanga et al., 2020). Citrulline supplementation during heat stress in chicks was found to be beneficial in reducing the rectal temperature down to the level of non-heat-stressed birds (Chowdhury et al., 2017). The regulation of core body temperature by citrulline is mediated through its effects on the secretion of inflammatory cytokines, initiating a neuroendocrine immunoregulatory cascade (Uyanga et al., 2022). Citrulline supplementation also promotes muscle protein synthesis by activating the mTORC1 pathway (Osowska et al., 2006; Le Plenier et al., 2011).

Though citrulline and GAA were able to replace arginine in low-protein poultry diets and demonstrate arginine-sparing effects (Esser et al., 2017), GAA was found to be less effective in replacing arginine (Dao et al., 2021b). However, the inclusion levels of the GAA and citrulline supplementation should also be considered; GAA at doses higher than 0.15% in poultry diets is demonstrated to have toxic effects on day 35 in Ross 308 male cockerels fed a low protein diet (Dao et al., 2021b), whereas doses ranging from 0.06%–0.12% promote growth and production in Ross 308 cockerels fed a basal diet on day 35 (Tossenberger et al., 2016). However, as indicated by several studies, the physiological effects of GAA and citrulline depend on factors such as the dose supplemented and the physiological status of the birds ((Ahmadipour et al., 2018; Uyanga et al., 2022)). Further studies are warranted to elucidate the biological events that underlie the response of poultry to citrulline and GAA supplementation.

## 7 Arginine and the macrophage dichotomy

Arginine and its metabolites serve as important mediators of several physiological processes affecting the health and production of poultry, extensively elaborated elsewhere (Ghamari Monavvar et al., 2020). This review focuses on the role of arginine and its metabolites in immune responses of poultry. Arginine is demonstrated to play a pivotal role in humoral and cell mediated immune responses in poultry (Ruan et al., 2020). Macrophages are professional cells of the innate immune system, which performs diverse functions. Macrophages are involved in the induction and resolution of an inflammatory reaction, tissue repair, and the activation of lymphocyte-mediated adaptive immune response (Miyashita et al., 2022). Macrophages adapt to the respective microenvironment and tissue niches in which they function. This adaptability enables macrophage polarization, which is the process by which macrophages mount a specific phenotypic and functional response to the microenvironmental stimuli encountered in a specific tissue (Sica and Mantovani, 2012). The polarization of macrophages is not fixed due to their multifaceted functions. Polarization of macrophages occurs in response to cell-to-cell interactions and cell-to-molecule interactions during an inflammatory response to maintain homeostasis. Macrophage polarization is regulated by arginine availability in the microenvironment and its metabolism by macrophages (Gharavi et al., 2022). Macrophage polarization can be categorized into M1 (classically activated macrophages or pro-inflammatory) and M2 (alternatively activated macrophages or anti-inflammatory) macrophages based on the arginine metabolism (Lumeng et al., 2007). M1 macrophages are induced by inflammatory mediators such as bacterial lipopolysaccharides and are characterized by the production of proinflammatory cytokines such as IFN-γ, IL-1β, IL-12, iNOS, TNF-α, and reactive oxygen species. M2 macrophages are induced by IL-4 and IL-13, which are Th2 cytokines important for the resolution of inflammation, tissue repair, and wound healing (Benoit et al., 2008). M1 macrophages are microbicidal and inflammatory, whereas M2 macrophages are anti-inflammatory and poor microbicides (Benoit et al., 2008).

Polarization of macrophages along the M1 and M2 axes occurs based on the activities of the arginine metabolizing enzymes NOS and arginase, respectively (Wentzel). Activation of macrophages by microbial products such as LPS, Th1 cytokines such as IL-1β, IFN-γ, or TNF-α, or stress such as hypoxia recruits macrophages to the M1 pathway and induces the expression of the iNOS gene, also known as macNOS because it was first discovered in activated macrophages (Molecular and epigenetic basis of, 2015). Furthermore, the M1 macrophages mediate their anti-microbial activity through the activation of the nicotinamide adenine dinucleotide phosphate (NADPH) oxidase system and the subsequent production of reactive oxygen species (Shapouri-Moghaddam et al., 2018). The availability of arginine and endogenous or pharmacological analogs of arginine can constrain NO synthesis. However, the effects of NO can be detrimental to the host tissues as well. The local concentrations of NO play an important role in cytotoxicity; under normal physiological conditions, NO is produced in picomolar quantities, whereas during inflammation, NO is produced in micromolar quantities (Abramson et al., 2001). Sustained increased production of NO damages the surrounding cells and tissues of the host as well, in addition to the pathogens (Abramson et al., 2001). Furthermore, NO causes lipid peroxidation and decreases the activity of serum antioxidants such as glutathione, causing oxidative stress (Qiu et al., 2019).

**FIGURE 4:**
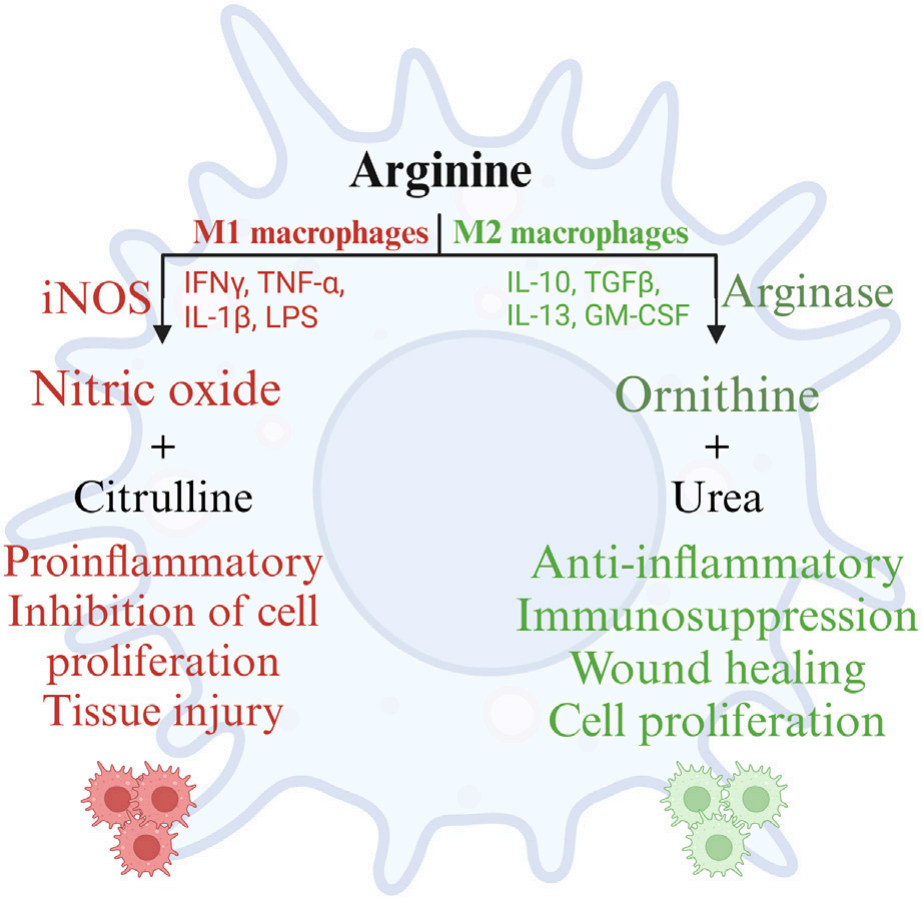
Metabolism of arginine in M1 and M2 macrophages. Created with BioRender.com (18 Nov 2022).

Unlike M1 macrophages, M2 macrophages metabolize arginine using the arginase pathway, which is stimulated by cytokines such as IL-4, IL-6, IL-10, IL-13, TGF-β, and other factors such as GM-CSF, PGE-2, cAMP, and catecholamines (Martí i Líndez and Reith, 2021). As the cytokines indicate, the arginase pathway in macrophages primarily promotes wound repair, matrix deposition, and healing (Martí i Líndez and Reith, 2021). These functions are mediated by the metabolism of L-arginine by arginase, yielding ornithine and urea. Ornithine is further metabolized by ornithine aminotransferase and ornithine decarboxylase to proline and polyamines, respectively. Proline is essential for collagen synthesis, while polyamines mediate diverse functions such as gene expression, translation, cell proliferation, cell growth, cell signaling, membrane stability, and cell death (Kusano et al., 2008; Li et al., 2022). Arginine supplementation thus reduces inflammation, intestinal injury, and oxidative stress, restoring intestinal homeostasis (Qiu et al., 2019). Arginase competes with NOSs for the common endogenous substrate L-arginine, preventing the overproduction of NO and associated tissue damage during prolonged inflammation. In short, during inflammation, the metabolism of arginine follows a biphasic pattern; initially, there will be a burst of microbicidal NO synthesis followed by an increase in the synthesis of ornithine, proline, and polyamines to promote the resolution of inflammation and wound healing (Martí i Líndez and Reith, 2021). However, iNOS can control arginase activity by generating hydroxy-L-arginine, an intermediate in NO synthesis, to inhibit arginase activity. Arginase, in turn, can deplete arginine in the extracellular milieu, thus regulating NO production (Choi et al., 2009). This chasm between the metabolic pathways of arginine in M1 and M2 macrophages is summarized in Figure 4.

Thus, arginine plays an important role in macrophage activation and function by serving as the sole endogenous substrate for the macrophage enzymes iNOS and arginase, mediating inflammation and resolution of inflammation, respectively.

## 8 Arginine and the gut microbiota

L-arginine is a metabolically versatile amino acid that serves as a source of carbon, nitrogen, and energy through different catabolic pathways in bacteria. Even though there is a vast diversity of gut microbiota, their metabolic redundancy and interaction with other microbiota species make their survival easier. The interaction between different species of gut microbiota and their metabolic products can have important implications for gut microbial composition, immune regulation, metabolism, and host health as well. It is thought that microbial amino acid utilization in the small intestine is for the synthesis of bacterial proteins. In contrast, amino acid catabolism dominates in the large intestine due to the lower availability of carbohydrates (Dai et al., 2011).

Arginine biosynthesis in bacteria occurs through the linear pathway, present in *E. coli*, or the recycling pathway, present in *Bacillus*. In the linear pathway, acetyl CoA condenses with glutamate to yield arginine through a series of eight steps, while in the recycling pathway, the acetyl group from acetylornithine is transferred to glutamate by the enzyme ornithine acetyltransferase. Both pathways are regulated by a negative feedback mechanism based on the concentration of arginine (Lu, 2006). The expression of these enzymes can be affected by other gut microbes and the intestinal compartment as well. In the case of *Lactobacillus plantarum*, it was observed that the expression of argininosuccinate synthase, an enzyme involved in arginine biosynthesis, increased significantly in mice’s gastrointestinal tract compared to its *in vitro* expression (Bron et al., 2004). Furthermore, the expression of argininosuccinate synthase is specifically induced in the small intestine of mice, compared to other sections of the gastrointestinal tract (Marco et al., 2007).

**FIGURE 5:**
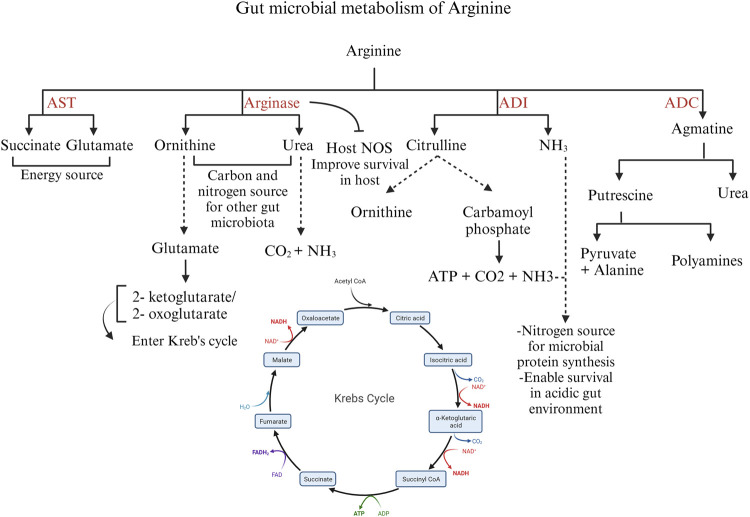
Different pathways of arginine catabolism in gut microbiota. Created with BioRender.com (16 September 2023).

The bacteria catabolize arginine via the arginase pathway, arginine deaminase pathway, arginine dehydrogenase/transaminase/oxidase pathway, and arginine succinyl transferase pathway (Lu, 2006). The expression of these enzymes can be affected by other gut microbes and the intestinal compartment. An outline of the bacterial catabolism by the four major enzymes is schematically represented in Figure 5.

The arginase pathway in bacteria is important to modulate the intracellular levels of arginine and ornithine in response to environmental conditions and physiological needs. Arginase expression or activity increases in the presence of exogenous arginine (Ide et al., 2020) In the arginase pathway, ornithine and urea are formed from arginine. Ornithine is transformed into glutamate by the enzymes ornithine aminotransferase and Δ-pyrroline-5-carboxylate dehydrogenase. Glutamate is converted into 2-ketoglutarate or 2-oxoglutarate, entering the Krebs cycle. Ornithine and urea generated by the arginase pathway can serve as carbon and nitrogen sources for other gut microbial species (Lu, 2006). In microorganisms producing the enzyme urease, the urea formed as a by-product is further hydrolyzed to carbon dioxide and ammonia, serving as a source of nitrogen (Hernández et al., 2021) Moreover, the arginase pathway serves as a survival mechanism for pathogenic bacteria such as *H. pylori*. *H. pylori* arginase inhibits the host NO synthesis by depleting the substrate arginine, thus promoting bacterial survival (Gobert et al., 2001).

The enzyme arginine decarboxylase (ADC) decarboxylates arginine to yield agmatine, which is further hydrolyzed to urea and putrescine by agmatinase. Putrescine, in turn, can be metabolized to pyruvate and alanine. Though putrescine can be metabolized to pyruvate, the ADC pathway is aimed at polyamine synthesis rather than an energy source (Schriek et al., 2007).

The arginine deiminase (ADI) pathway is induced in bacteria under microaerobic and anaerobic conditions. The ADI gene is expressed by several microbes such as *Bacillus licheniformis*, *Clostridium perfringens*, and *Enterococcus faecalis* (Lu, 2006). ADI deiminates arginine to citrulline and ammonia. Citrulline is further converted into ornithine and carbamoyl phosphate by the enzyme ornithine transcarbamoylase. Carbamate kinase mediates ATP production from carbamoyl phosphate with carbon dioxide and ammonia as by-products. Thus, the ADI pathway produces ATP for energy and ammonia as a nitrogen source for the bacteria (Lu, 2006). In addition, ammonia aids in the survival of pathogenic bacteria, such as *C. perfringens,* under acidic conditions (Myers et al., 2006) In bacteria expressing arginase and ADI enzymes, the arginase pathway is predominant under aerobic conditions, whereas the ADI pathway predominates during anaerobic conditions (Hernández et al., 2021).

The arginine succinyl transferase (AST) enzyme mediates the transfer of the succinyl group from succinyl CoA to arginine and further the production of succinate and glutamate through a series of chemical reactions. The AST pathway is the preferred pathway for arginine catabolism in *Pseudomonas* (Hernández et al., 2021); however, in *E. coli*, the AST pathway is stimulated during carbon starvation, when the priority is cell survival using arginine as a nitrogen source. In some *Pseudomonas* species, such as *P. aeruginosa*, arginine transaminase supplements succinate production under aerobic conditions. Arginine transaminase uses ketoarginine as a substrate, produced by L-arginine: pyruvate transaminase, arginine oxidase, and arginine dehydrogenase (Li and Lu, 2009). Thus, arginine functions as a microbial energy source, governs the expression of bacterial virulence genes, and actively modulates the host’s immune response to the gut microbiota (Choi et al., 2012). Despite these known roles, understanding the specific impact of arginine on poultry gut microbiota and enteric pathogens, and its precise involvement in shaping the pathogenesis of enteric diseases requires further elucidation.

## 9 Arginine and necrotic enteritis

The use of arginine in low-protein diets to improve gut health is recently being investigated in poultry, especially in relation to the control of necrotic enteritis (Zhang et al., 2019; Dao et al., 2022a; Dao et al., 2022b; Dao et al., 2022c). Arginine can modulate the birds’ innate and adaptive immune responses to the *C. perfringens* challenge (Zhang et al., 2019). Arginine exerts its effect primarily through the metabolites NO and ornithine, which further take part in downstream reactions or are metabolized to bioactive molecules that take part in inflammation or the resolution of inflammation (Zhang et al., 2019). Arginine modulates macrophage polarization towards the M1 or M2 pathway, significantly affecting the innate immune response to pathogens, including *C. perfringens* (Kim et al., 2022). Apart from its role in shaping the innate immune response, arginine is important in regulating the adaptative immune response to necrotic enteritis and alleviating inflammatory damage caused by necrotic enteritis (Zhang et al., 2019).

**FIGURE 6:**
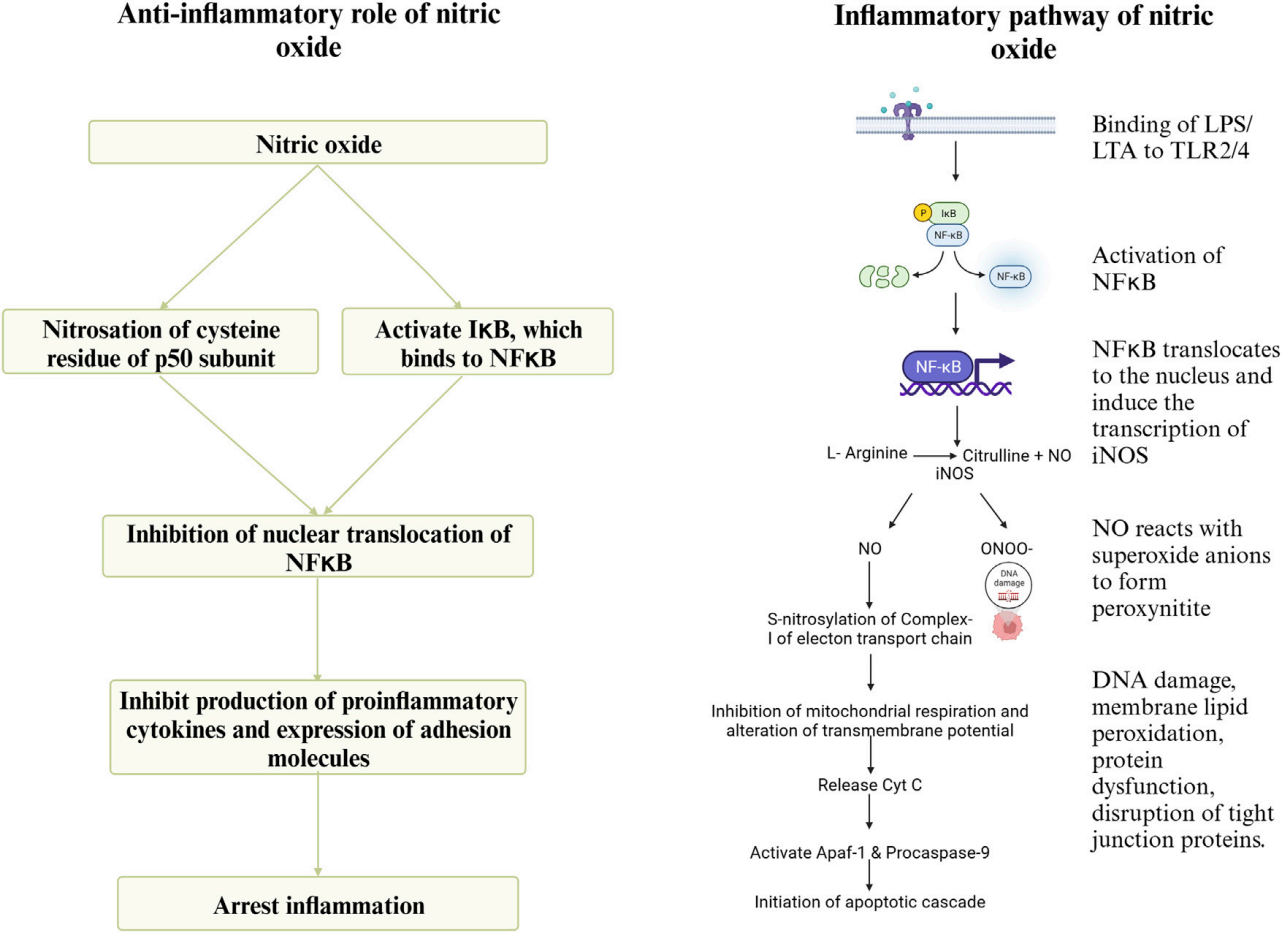
Created with BioRender.com (September 15, 2023).

During infection by *C. perfringens*, the Toll-like Receptor (TLR)- 2 recognizes lipoteichoic acid in the cell wall of *C. perfringens* and initiates downstream signaling, leading to the activation of the transcription factor NFκB, which translocates to the nucleus and induces the expression of iNOS (Korhonen et al., 2002). Inducible NOS converts arginine to NO. Nitric oxide (NO) reacts with superoxide anions to form peroxynitrite (ONOO-), which causes DNA damage, membrane lipid peroxidation, protein dysfunction by nitration of tyrosine residues, and the disruption of tight junction proteins (Potoka et al., 2002), thus increasing intestinal permeability (Korhonen et al., 2002). nNOS and eNOS are expressed and produce picomolar quantities of NO. In contrast, iNOS, expressed in response to inflammation, produces micromolar quantities of NO (Nathan and Xie, 1994). The apoptotic effect of sustained high concentrations of NO and peroxynitrite is due to the inhibition of mitochondrial respiration by S-nitrosylation of complex-I. Additionally, the inhibition of mitochondrial respiration can decrease the transmembrane potential, leading to the release of cytochrome-c, which interacts with the cytoplasmic apoptosis activating factor-1 (Apaf-1) and procaspase-9 initiating the apoptotic cascade (Potoka et al., 2002). Previous research findings indicate an upregulation of NOS gene expression, increased gut permeability, and decreased expression of tight junction proteins and nutrient transporters during necrotic enteritis (Dao et al., 2022d). However, NO can play a significant role in the resolution of enteritis as well. NO can cause nitrosation of the p50 subunit of NFκB or activate IκB, the inhibitor protein for NFκB, and thus regulate the production of proinflammatory cytokines (McCafferty et al., 1997). Sustained overproduction of NO reduces the circulating levels of IL-6 and TNF, downregulating the expression of adhesion molecules. This, in turn, will reduce neutrophil adhesion in inflammatory sites and host tissue damage (Muñoz-Fernández and Fresno, 1998). A schematic representation of the pathway through which arginine mediates the proinflammatory and anti-inflammatory roles during necrotic enteritis is shown in Figure 6.

**FIGURE 7:**
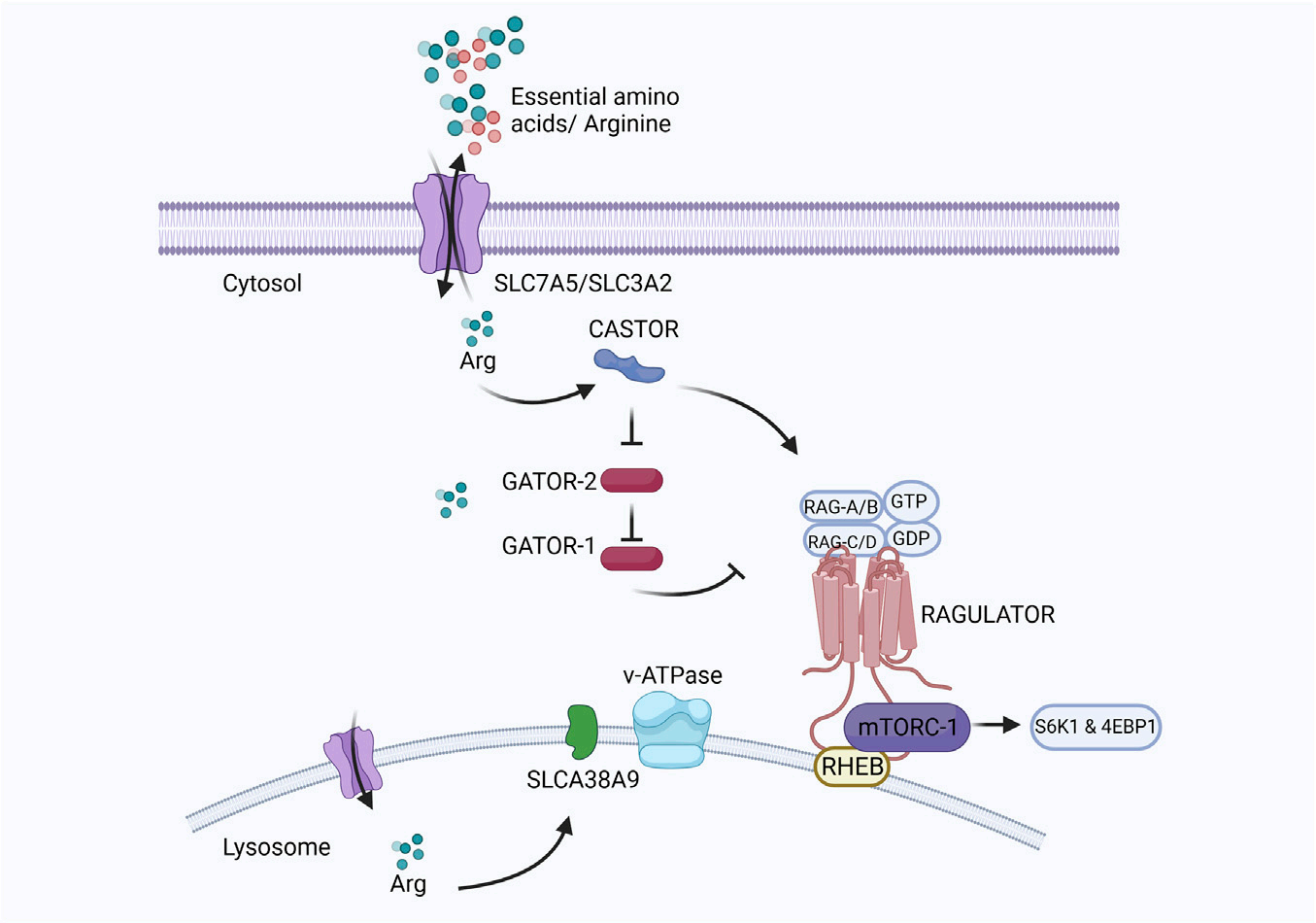
Created with BioRender.com (September 18, 2023).

At the cellular level, the mechanistic target of rapamycin complex I (mTORC1) regulates eukaryotic cell metabolism, growth, proliferation, and survival in response to environmental signals such as nutrients and growth factors (Cummings and Lamming, 2017). Under adequate arginine conditions, the cytosolic arginine sensor cellular arginine sensor for mTORC1 (CASTOR) interacts with the GAP activity toward Rags (GATOR), a negative regulator of mTORC1. GATOR2 lies upstream of GATOR1 and suppresses the RagA/B GTPase-Activating Protein (GAP) activity of GATOR1 under sufficient arginine conditions. Activated RagA/B binds GTP, and RagC/D binds GDP and is anchored to the lysosome by the Ragulator protein (Wolfson et al., 2016; Jung et al., 2019). Rag proteins mediate lysosomal recruitment of mTORC1, which is subsequently activated by Ras homologs enriched in the brain (Rheb) present on the lysosomal membrane. Activation of mTORC1 leads to the phosphorylation of S6 kinase-1 (S6K1) and eukaryotic translation initiation factor 4E-binding protein-1 (4EBP1), which stimulates protein translation and cell growth (Wolfson and Sabatini, 2017). A diagrammatic representation of the regulatory pathway of mTORC1 in cells is presented in Figure 7.

L-arginine is one of the three amino acids (arginine, glutamine, and leucine) that can directly regulate mTORC1 activation and, thus, cell proliferation and apoptosis. Arginine interacts with the transcriptional regulators in the mTOR pathway, enhancing T-cell survival and memory T-cell formation (Geiger et al., 2016). Intracellular arginine availability is thus an important determinant of T-cell function. Arginine metabolism by arginase depletes arginine in the microenvironment, causing T-cell hypo responsiveness. Arginine depletion inhibits proliferation, downregulates the expression of activation markers, and decreases cytokine production in T-cells. Arginase-mediated arginine starvation arrests the cell cycle at the G_0_-G_1_ phase (Rodriguez et al., 2007). Arginine availability in cells regulates T-cell survival and activity by producing NO. NO exerts proapoptotic effects on T-cells by regulating the intracellular signaling protein expression (Choi et al., 2009).

Arginine supplementation during necrotic enteritis depletes the arginine degradation pathways in gut microbiota, including *C. perfringens*, sparing arginine for T-cell proliferation and function and thus inhibiting disease progression (Dao et al., 2022c) T-lymphocytes, particularly Th1 cells, play an important role in pathogen clearance and adaptive immunity during necrotic enteritis (Fathima et al., 2022). Dietary arginine supplementation increases the T-cell population and promotes T-cell activation and survival (Kishton et al., 2016), thus helping faster recovery. Naïve or quiescent T-cells use oxidative phosphorylation for their energy supply and require little nutrients, whereas activated T-cells rely on glycolytic and glutaminolytic pathways for their energy needs and consume large amounts of amino acids, glucose, and fatty acids in the process (Geiger et al., 2016). Activated T-cells heavily consume arginine, causing a marked drop in serum arginine levels. This drop in serum arginine is observed during poultry coccidiosis (Allen and Fetterer, 2000), an important predisposing factor for necrotic enteritis. This can be due to the increased requirement for nutrients to enhance the survival of T-cells during infection and the development of memory T-cells during recovery. Further, this can be correlated with the increased proliferation of intestinal epithelial cells, protein synthesis, and reduced intestinal epithelial cell damage during arginine supplementation *in vitro* (Tan et al., 2010). L-arginine supplementation upregulates the mRNA expression of the tight junction proteins ZO-1, claudin-1, and occludin, resulting in reduced intestinal injury, improved intestinal permeability, and increased villus height: crypt ratio in poultry. Arginine supplementation also inhibits *C. perfringens* colonization, reduces the gross pathology associated with necrotic enteritis and hepatic translocation of *C. perfringens*, improves intestinal absorption and barrier function, and attenuates intestinal inflammatory responses (Zhang et al., 2017; Zhang et al., 2019).

## 10 Emerging trends and future prospects of arginine in poultry production

Arginine is a functional amino acid of paramount importance in ensuring the health and wellbeing of poultry. It assumes a multitude of critical roles within avian physiology, encompassing functions such as growth, metabolism, immune response, and gut microbial homeostasis. Together, these interconnected aspects highlight the pivotal role of arginine in shaping the nutritional status, immune response, and overall wellbeing of poultry. Arginine offers a promising avenue for improving poultry health and the sustainability of the poultry industry. Though existing research acknowledges the importance of arginine in poultry nutrition beyond protein synthesis, further research is warranted to investigate the optimum levels of arginine and arginine metabolites in poultry diets under different production systems, stages of production, breeds, and physiological states. The potential role of arginine in preventing enteric diseases such as coccidiosis and necrotic enteritis in poultry has been explored to some extent. Still, it offers wider arenas for further understanding arginine’s specific mode of action during these disease processes. The possible modulation of gut microbiota by arginine and its association with disease incidence, severity, and gut health during enteric diseases is poorly investigated in poultry. An understanding of the impact of arginine on gut barrier function, immune response, and gut microbial homeostasis can give insights into the potential use of arginine for improving the health and production in poultry.

## References

[B1] 2022-Cobb500-Broiler-Performance-Nutrition-Supplement (2022) 2022-Cobb500-Broiler-Performance-Nutrition-Supplement.

[B2] Abramson S. B. Amin A. R. Clancy R. M. Attur M. (2001). The role of nitric oxide in tissue destruction. Best Pract. Res. Clin. Rheumatology 15 (5), 831–845. 10.1053/berh.2001.0196 11812024

[B3] Adams S. Che D. Qin G. Farouk M. H. Hailong J. Rui H. (2019). Novel biosynthesis, metabolism and physiological functions of L-homoarginine. Curr. Protein Peptide Sci. 20 (2), 184–193. 10.2174/1389203719666181026170049 30370846

[B4] Ahmadipour B. Khajali F. Sharifi M. R. (2018). Effect of guanidinoacetic acid supplementation on growth performance and gut morpholog yin broiler chickens. Poult. Sci. J. 6 (1), 19–24. 10.2141/jpsa.0170044 PMC6756380 32055157

[B5] Akasaka N. Fujiwara S. (2020). The therapeutic and nutraceutical potential of agmatine, and its enhanced production using Aspergillus oryzae. Amino Acids 52 (2), 181–197. 10.1007/s00726-019-02720-7 30915570

[B6] Al-Daraji H. J. Salih A. M. (2012). Effect of dietary L-arginine on productive performance of broiler chickens. Pak. J. Nutr. 11 (3), 252–257. 10.3923/pjn.2012.252.257

[B7] Allen P. C. Fetterer R. H. (2000). Effect of Eimeria acervulina infections on plasma L-arginine. Poult. Sci. 79 (10), 1414–1417. 10.1093/ps/79.10.1414 11055846

[B8] Almquist H. J. Mecchi E. Kratzer F. H. (1941). Creatine formation in the chick. J. Biol. Chem. 141 (2), 365–373. 10.1016/s0021-9258(18)72782-8

[B9] Amiri M. Ghasemi H. A. Hajkhodadadi I. Farahani A. H. K. (2019). Efficacy of guanidinoacetic acid at different dietary crude protein levels on growth performance, stress indicators, antioxidant status, and intestinal morphology in broiler chickens subjected to cyclic heat stressficacy of guanidinoacetic acid at different dietary crude protein levels on growth performance, stress indicators, antioxidant status, and intestinal morphology in broiler chickens subjected to cyclic heat stress. Anim. Feed Sci. Technol. 254, 114208. 10.1016/j.anifeedsci.2019.114208

[B10] Anderson J. O. Combs G. F. (1952). Effect of single amino acid excesses on glucose metabolism and chick growth, as influenced by the dietary amino acid balance. J. Nutr. 46 (2), 161–170. 10.1093/jn/46.2.161 14908690

[B11] Applegate T. J. Angel R. (2014). Nutrient requirements of poultry publication: history and need for an update. J. Appl. Poult. Res. 23 (3), 567–575. 10.3382/japr.2014-00980

[B12] Application of nutritional immunology (2022). “Application of nutritional immunology in the mitigation of economic and production losses in the poultry industry associated with food-borne pathogens, coccidiosis, and necrotic enteritis,” in Proceedings of the Arkansas Nutrition Conference.

[B13] Arginine sparing potential of guanidinoacetic acid in broiler nutrition (2018). “Arginine sparing potential of guanidinoacetic acid in broiler nutrition,” in PSA Latin American Scientific Conference.

[B14] Austic R. E. Nesheim M. C. (1970). Role of kidney arginase in variations of the arginine requirement of chicks. J. Nutr. 100 (7), 855–867. 10.1093/jn/100.7.855 5457056

[B15] Ball R. O. Urschel K. L. Pencharz P. B. (2007). Nutritional consequences of interspecies differences in arginine and lysine metabolism. J. Nutr. 137 (6), 1626S–1641S. 10.1093/jn/137.6.1626S 17513439

[B16] Bell E. A. (2003). Nonprotein amino acids of plants: significance in medicine, nutrition, and agriculture. J. Agric. Food Chem. 51 (10), 2854–2865. 10.1021/jf020880w 12720365

[B17] Benoit M. Desnues B. Mege J. (2008). Macrophage polarization in bacterial infections. J. Immunol. 181 (6), 3733–3739. 10.4049/jimmunol.181.6.3733 18768823

[B18] Bowen O. T. Erf G. F. Chapman M. E. Wideman R. F. Jr (2007). Plasma nitric oxide concentrations in broilers after intravenous injections of lipopolysaccharide or microparticles. Poult. Sci. 86 (12), 2550–2554. 10.3382/ps.2007-00288 18029801

[B19] Brake J. Balnave D. Dibner J. J. (1998). Optimum dietary arginine: lysine ratio for broiler chickens is altered during heat stress in association with changes in intestinal uptake and dietary sodium chloride. Br. Poult. Sci. 39 (5), 639–647. 10.1080/00071669888511 9925317

[B20] Bröer S. Fairweather S. J. (2018). Amino acid transport across the mammalian intestine. Compr. Physiol. 9 (1), 343–373. 10.1002/cphy.c170041 30549024

[B21] Bron P. A. Grangette C. Mercenier A. De Vos W. M. Kleerebezem M. (2004). Identification of Lactobacillus plantarum genes that are induced in the gastrointestinal tract of mice. J. Bacteriol. 186 (17), 5721–5729. 10.1128/JB.186.17.5721-5729.2004 15317777 PMC516819

[B22] Chamruspollert M. Pesti G. M. Bakalli R. I. (2002). Dietary interrelationships among arginine, methionine, and lysine in young broiler chicks. Br. J. Nutr. 88 (6), 655–660. 10.1079/BJN2002732 12493087

[B23] Choi B. S. Martinez-Falero I. C. Corset C. Munder M. Modolell M. Müller I. (2009). Differential impact of L-arginine deprivation on the activation and effector functions of T cells and macrophages. J. Leucocyte Biol. 85 (2), 268–277. 10.1189/jlb.0508310 PMC2642643 19008294

[B24] Choi Y. Choi J. Groisman E. A. Kang D. Shin D. Ryu S. (2012). Expression of STM4467-encoded arginine deiminase controlled by the STM4463 regulator contributes to *Salmonella enterica* serovar Typhimurium virulence. Infect. Immun. 80 (12), 4291–4297. 10.1128/IAI.00880-12 23006851 PMC3497419

[B25] Chowdhury V. S. Han G. Bahry M. A. Tran P. V. Do P. H. Yang H. (2017). L-Citrulline acts as potential hypothermic agent to afford thermotolerance in chicks. J. Therm. Biol. 69, 163–170. 10.1016/j.jtherbio.2017.07.007 29037378

[B26] Closs E. I. Mann G. E. (2000). Membrane transport of L-arginine and cationic amino acid analogs. Nitric Oxide, 225–241. 10.1016/b978-012370420-7/50015-0

[B27] Closs E. I. Simon A. Vékony N. Rotmann A. (2004). Plasma membrane transporters for arginine. J. Nutr. 134 (10), 2752S–2767S. 10.1093/jn/134.10.2752S 15465780

[B28] Corzo A. Lee J. Vargas J. I. Silva M. Pacheco W. J. (2021). Determination of the optimal digestible arginine to lysine ratio in Ross 708 male broilers. J. Appl. Poult. Res. 30 (1), 100136. 10.1016/j.japr.2020.100136

[B29] Cummings N. E. Lamming D. W. (2017). Regulation of metabolic health and aging by nutrient-sensitive signaling pathways. Mol. Cell Endocrinol. 455, 13–22. 10.1016/j.mce.2016.11.014 27884780 PMC5440210

[B30] Dai Z. Wu G. Zhu W. (2011). Amino acid metabolism in intestinal bacteria: links between gut ecology and host health. Front. Bioscience-Landmark 16 (5), 1768–1786. 10.2741/3820 21196263

[B31] Dao H. T. Clay J. W. Sharma N. K. Bradbury E. J. Swick R. A. (2022b). Effects of l-arginine and l-citrulline supplementation in reduced protein diets on cecal fermentation metabolites of broilers under normal, cyclic warm temperature and necrotic enteritis challenge. Livest. Sci. 257, 104826. 10.1016/j.livsci.2022.104826

[B32] Dao H. T. Sharma N. K. Barekatain R. Kheravii S. K. Bradbury E. J. Wu S. (2022c). Supplementation of reduced protein diets with. Animal Prod. Sci. 62, 1250–1265. 10.1071/an21394

[B33] Dao H. T. Sharma N. K. Bradbury E. J. Swick R. A. (2021a). Effects of L-arginine and L-citrulline supplementation in reduced protein diets for broilers under normal and cyclic warm temperature. Anim. Nutr. 7 (4), 927–938. 10.1016/j.aninu.2020.12.010 34703910 PMC8526778

[B34] Dao H. T. Sharma N. K. Bradbury E. J. Swick R. A. (2021b). Response of meat chickens to different sources of arginine in low-protein diets. J. Anim. Physiol. Anim. Nutr. 105 (4), 731–746. 10.1111/jpn.13486 33410556

[B35] Dao H. T. Sharma N. K. Daneshmand A. Kumar A. Bradbury E. J. Wu S. (2022a). Supplementation of reduced protein diets with. Animal Prod. Sci. 62, 1236–1249. 10.1071/an21393

[B36] Dao H. T. Sharma N. K. Kheravii S. K. Bradbury E. J. Wu S. Swick R. A. (2022d). Supplementation of reduced protein diets with. Animal Prod. Sci. 62 (13), 1266–1279. 10.1071/an21395

[B37] DeGroot A. A. Braun U. Dilger R. N. (2018). Efficacy of guanidinoacetic acid on growth and muscle energy metabolism in broiler chicks receiving arginine-deficient diets. Poult. Sci. 97 (3), 890–900. 10.3382/ps/pex378 29294127

[B38] D'mello J. (2003a). Adverse effects of amino acids. Amino acids animal Nutr. 2, 125–142. 10.1079/9780851996547.0125

[B39] D'mello J. (2003b). Amino acids as multifunctional molecules. Amino acids in animal nutrition. (Wallingford UK: Cabi Publishing). 1–14.

[B40] D’mello J. Lewis D. (1970). Amino acid interactions in chick nutrition: I. The interrelationship between lysine and arginine. Br. Poult. Sci. 11 (3), 299–311. 10.1080/00071667008415820 5433121

[B41] El-Hattab A. W. Emrick L. T. Craigen W. J. Scaglia F. (2012). Citrulline and arginine utility in treating nitric oxide deficiency in mitochondrial disorders. Mol. Genet. Metab. 107 (3), 247–252. 10.1016/j.ymgme.2012.06.018 22819233

[B42] Esser A. Gonçalves D. Rorig A. Cristo A. B. Perini R. Fernandes J. (2017). Effects of guanidionoacetic acid and arginine supplementation to vegetable diets fed to broiler chickens subjected to heat stress before slaughter. Braz. J. Poult. Sci. 19, 429–436. 10.1590/1806-9061-2016-0392

[B43] Fagundes N. S. Milfort M. C. Williams S. M. Da Costa M. J. Fuller A. L. Menten J. F. (2020). Dietary methionine level alters growth, digestibility, and gene expression of amino acid transporters in meat-type chickens. Poult. Sci. 99 (1), 67–75. 10.3382/ps/pez588 32416854 PMC7587823

[B44] Fathima S. Hakeem W. G. A. Shanmugasundaram R. Selvaraj R. K. (2022). Necrotic enteritis in broiler chickens: a review on the pathogen, pathogenesis, and prevention. Microorganisms 10 (10), 1958. 10.3390/microorganisms10101958 36296234 PMC9610872

[B45] Faure M. Choné F. Mettraux C. Godin J. Béchereau F. Vuichoud J. (2007). Threonine utilization for synthesis of acute phase proteins, intestinal proteins, and mucins is increased during sepsis in rats. J. Nutr. 137 (7), 1802–1807. 10.1093/jn/137.7.1802 17585034

[B46] Fletcher M. T. Al Jassim R. A. Cawdell-Smith A. J. (2015). The occurrence and toxicity of indospicine to grazing animals. Agriculture 5 (3), 427–440. 10.3390/agriculture5030427

[B47] Fosoul SSAS Azarfar A. Gheisari A. Khosravinia H. (2018). Energy utilisation of broiler chickens in response to guanidinoacetic acid supplementation in diets with various energy contents. Br. J. Nutr. 120 (2), 131–140. 10.1017/S0007114517003701 29690949

[B48] Geiger R. Rieckmann J. C. Wolf T. Basso C. Feng Y. Fuhrer T. (2016). L-arginine modulates T cell metabolism and enhances survival and anti-tumor activity. Cell 167 (3), 829–842. 10.1016/j.cell.2016.09.031 27745970 PMC5075284

[B49] Ghamari Monavvar H. Moghaddam G. Ebrahimi M. (2020). A review on the effect of arginine on growth performance, meat quality, intestine morphology, and immune system of broiler chickens. Iran. J. Appl. Animal Sci. 10 (4), 587–594.

[B50] Gharavi A. T. Hanjani N. A. Movahed E. Doroudian M. (2022). The role of macrophage subtypes and exosomes in immunomodulation. Cell Mol. Biol. Lett. 27 (1), 83. 10.1186/s11658-022-00384-y 36192691 PMC9528143

[B51] Gobert A. P. McGee D. J. Akhtar M. Mendz G. L. Newton J. C. Cheng Y. (2001). *Helicobacter pylori* arginase inhibits nitric oxide production by eukaryotic cells: a strategy for bacterial survival. Proc. Natl. Acad. Sci. 98 (24), 13844–13849. 10.1073/pnas.241443798 11717441 PMC61129

[B52] Grishin D. V. Zhdanov D. D. Pokrovskaya M. V. Sokolov N. N. (2020). D-amino acids in nature, agriculture and biomedicine. All Life 13 (1), 11–22. 10.1080/21553769.2019.1622596

[B53] Haghikia A. Yanchev G. R. Kayacelebi A. A. Hanff E. Bledau N. Widera C. (2017). The role of L-arginine/L-homoarginine/nitric oxide pathway for aortic distensibility and intima-media thickness in stroke patients. Amino Acids 49 (6), 1111–1121. 10.1007/s00726-017-2409-2 28285332

[B54] Ham D. J. Gleeson B. G. Chee A. Baum D. M. Caldow M. K. Lynch G. S. (2015). L-Citrulline protects skeletal muscle cells from cachectic stimuli through an iNOS-dependent mechanism. PLoS One 10 (10), e0141572. 10.1371/journal.pone.0141572 26513461 PMC4625972

[B55] Hegarty M. P. Pound A. W. (1970). Indospicine, a hepatotoxic amino acid from Indigofera spicat A: isolation, structure, and biological studies. Aust. J. Biol. Sci. 23 (4), 831–842. 10.1071/bi9700831

[B56] Hernández V. M. Arteaga A. Dunn M. F. (2021). Diversity, properties and functions of bacterial arginases. FEMS Microbiol. Rev. 45 (6), fuab034. 10.1093/femsre/fuab034 34160574

[B57] Ide A. A. Hernández V. M. Medina-Aparicio L. Carcamo-Noriega E. Girard L. Hernández-Lucas I. (2020). Genetic regulation, biochemical properties and physiological importance of arginase from Sinorhizobium meliloti. Microbiology 166 (5), 484–497. 10.1099/mic.0.000909 32216867

[B58] Investigation of protein quality (1968). “Investigation of protein quality--ileal recovery of amino acids,” in Federation Proceedings. 5691708

[B59] Jahanian R. (2009). Immunological responses as affected by dietary protein and arginine concentrations in starting broiler chicks. Poult. Sci. 88 (9), 1818–1824. 10.3382/ps.2008-00386 19687265

[B60] Jimoh O. A. Akinola M. O. Oyeyemi B. F. Oyeyemi W. A. Ayodele S. O. Omoniyi I. S. (2021). Potential of watermelon (*Citrullus lanatus*) to maintain oxidative stability of rooster semen for artificial insemination. J. Animal Sci. Technol. 63 (1), 46–57. 10.5187/jast.2021.e21 PMC7882837 33987583

[B61] Johnson S. L.-C. (2017). United States: US food and drug administration FDA. 2.

[B62] Jones J. D. Petersburg S. J. Burnett P. C. (1967). The mechanism of the lysine-arginine antagonism in the chick: effect of lysine on digestion, kidney arginase, and liver transamidinase. J. Nutr. 93 (1), 103–116. 10.1093/jn/93.1.103 6053753

[B63] Jung J. W. Macalino S. J. Y. Cui M. Kim J. E. Kim H. Song D. (2019). Transmembrane 4 L six family member 5 senses arginine for mTORC1 signaling. Cell Metab. 29 (6), 1306–1319. 10.1016/j.cmet.2019.03.005 30956113

[B64] Kadirvel R. Kratzer F. H. (1974). Uptake of L-arginine and L-lysine by the small intestine and its influence on arginine-lysine antagonism in chicks. J. Nutr. 104 (3), 339–343. 10.1093/jn/104.3.339 4811983

[B65] Kaul S. Sharma S. S. Mehta I. K. (2008). Free radical scavenging potential of L-proline: evidence from *in vitro* assays. Amino Acids 34 (2), 315–320. 10.1007/s00726-006-0407-x 17086481

[B66] Khajali F. Lemme A. Rademacher-Heilshorn M. (2020). Guanidinoacetic acid as a feed supplement for poultry. Worlds Poult. Sci. J. 76 (2), 270–291. 10.1080/00439339.2020.1716651

[B67] Khajali F. Wideman R. F. (2010). Dietary arginine: metabolic, environmental, immunological and physiological interrelationships. Worlds Poult. Sci. J. 66 (4), 751–766. 10.1017/s0043933910000711

[B68] Kidd M. T. Kerr B. J. Anthony N. B. (1997). Dietary interactions between lysine and threonine in broilers. Poult. Sci. 76 (4), 608–614. 10.1093/ps/76.4.608 9106889

[B69] Kim Y. J. Lee J. Lee J. J. Jeon S. M. Silwal P. Kim I. S. (2022). Arginine-mediated gut microbiome remodeling promotes host pulmonary immune defense against nontuberculous mycobacterial infection. Gut microbes 14 (1), 2073132. 10.1080/19490976.2022.2073132 35579969 PMC9116420

[B70] Kishton R. J. Sukumar M. Restifo N. P. (2016). Arginine arms T cells to thrive and survive. Cell metab. 24 (5), 647–648. 10.1016/j.cmet.2016.10.019 27829132 PMC6327309

[B71] Kodambashi Emami N. Golian A. Rhoads D. D. Danesh Mesgaran M. (2017). Interactive effects of temperature and dietary supplementation of arginine or guanidinoacetic acid on nutritional and physiological responses in male broiler chickens. Br. Poult. Sci. 58 (1), 87–94. 10.1080/00071668.2016.1257779 28052696

[B72] Korhonen R. Korpela R. Moilanen E. (2002). Signalling mechanisms involved in the induction of inducible nitric oxide synthase by Lactobacillus rhamnosus GG, endotoxin, and lipoteichoic acid. Inflammation 26 (5), 207–214. 10.1023/a:1019720701230 12238563

[B73] Kusano T. Berberich T. Tateda C. Takahashi Y. (2008). Polyamines: essential factors for growth and survival. Planta 228, 367–381. 10.1007/s00425-008-0772-7 18594857

[B74] Lehninger A. L. Nelson D. L. Cox M. M. (2005). Lehninger principles of biochemistry. Macmillan.

[B75] Lemme A. Ringel J. Sterk A. Young J. F. (2007). “Supplemental guanidino acetic acid affects energy metabolism of broilers,” in Proceedings 16th European Symposium on Poultry Nutrition (Strasbourg France).

[B76] Leong H. X. Simkevich C. Lesieur-Brooks A. Lau B. W. Fugere C. Sabo E. (2006). Short-term arginine deprivation results in large-scale modulation of hepatic gene expression in both normal and tumor cells: microarray bioinformatic analysis. Nutr. metabolism 3, 37–13. 10.1186/1743-7075-3-37 PMC1613245 16961918

[B77] Le Plenier S. Walrand S. Cynober L. Moinard C. (2011). OP049 direct action of citrulline on muscle protein synthesis: role of the mtorc1 pathway. Clin. Nutr. Suppl. 1 (6), 20. 10.1016/s1744-1161(11)70049-7

[B78] Li C. Lu C. (2009). Arginine racemization by coupled catabolic and anabolic dehydrogenases. Proc. Natl. Acad. Sci. 106 (3), 906–911. 10.1073/pnas.0808269106 19139398 PMC2630070

[B79] Li Z. Wang L. Ren Y. Huang Y. Liu W. Lv Z. (2022). Arginase: shedding light on the mechanisms and opportunities in cardiovascular diseases. Cell Death Discov. 8 (1), 413. 10.1038/s41420-022-01200-4 36209203 PMC9547100

[B80] Lima M. B. Sakomura N. K. Silva E. P. Leme B. B. Malheiros E. B. Peruzzi N. J. (2020). Arginine requirements for maintenance and egg production for broiler breeder hens. Anim. Feed Sci. Technol. 264, 114466. 10.1016/j.anifeedsci.2020.114466

[B81] Linder C. H. (2016). No title. Biochemical and functional properties of mammalian bone alkaline phosphatase isoforms during osteogenesis.

[B82] Liu G. Ajao A. M. Shanmugasundaram R. Taylor J. Ball E. Applegate T. J. (2023). The effects of arginine and branched-chain amino acid supplementation to reduced-protein diet on intestinal health, cecal short-chain fatty acid profiles, and immune response in broiler chickens challenged with Eimeria spp. Poult. Sci. 102 (7), 102773. 10.1016/j.psj.2023.102773 37236037 PMC10232892

[B83] Lu C. (2006). Pathways and regulation of bacterial arginine metabolism and perspectives for obtaining arginine overproducing strains. Appl. Microbiol. Biotechnol. 70, 261–272. 10.1007/s00253-005-0308-z 16432742

[B84] Lumeng C. N. Bodzin J. L. Saltiel A. R. (2007). Obesity induces a phenotypic switch in adipose tissue macrophage polarization. J. Clin. Invest 117 (1), 175–184. 10.1172/JCI29881 17200717 PMC1716210

[B85] MacMicking J. Xie Q. Nathan C. (1997). Nitric oxide and macrophage function. Annu. Rev. Immunol. 15, 323–350. 10.1146/annurev.immunol.15.1.323 9143691

[B86] Majdeddin M. Braun U. Lemme A. Golian A. Kermanshahi H. De Smet S. (2020). Guanidinoacetic acid supplementation improves feed conversion in broilers subjected to heat stress associated with muscle creatine loading and arginine sparing. Poult. Sci. 99 (9), 4442–4453. 10.1016/j.psj.2020.05.023 32867988 PMC7598026

[B87] Marco M. L. Bongers R. S. de Vos W. M. Kleerebezem M. (2007). Spatial and temporal expression of Lactobacillus plantarum genes in the gastrointestinal tracts of mice. Appl. Environ. Microbiol. 73 (1), 124–132. 10.1128/AEM.01475-06 17071785 PMC1797133

[B88] Martí i Líndez A. Reith W. (2021). Arginine-dependent immune responses. Cell. Mol. Life Sci. 78 (13), 5303–5324. 10.1007/s00018-021-03828-4 34037806 PMC8257534

[B89] Maynard C. W. Kidd M. T. (2022). Broiler amino acid research: then and now. Broiler Industry: IntechOpen.

[B90] McCafferty D. Mudgett J. S. Swain M. G. Kubes P. (1997). Inducible nitric oxide synthase plays a critical role in resolving intestinal inflammation. Gastroenterology 112 (3), 1022–1027. 10.1053/gast.1997.v112.pm9041266 9041266

[B91] McNab J. M. (1994). Amino acid digestibility and availability studies with poultry.

[B92] Meyer L. E. Machado L. B. Santiago A. P. S. da-Silva W. S. De Felice F. G. Holub O. (2006). Mitochondrial creatine kinase activity prevents reactive oxygen species generation: antioxidant role of mitochondrial kinase-dependent ADP re-cycling activity. J. Biol. Chem. 281 (49), 37361–37371. 10.1074/jbc.M604123200 17028195

[B93] Miri B. Ghasemi H. A. Hajkhodadadi I. Farahani A. H. K. (2022). Effects of low eggshell temperatures during incubation, in ovo feeding of L-arginine, and post-hatch dietary guanidinoacetic acid on hatching traits, performance, and physiological responses of broilers reared at low ambient temperature. Poult. Sci. 101 (1), 101548. 10.1016/j.psj.2021.101548 34823169 PMC8626698

[B94] Miska K. B. Fetterer R. H. (2017). The mRNA expression of amino acid and sugar transporters, aminopeptidase, as well as the di-and tri-peptide transporter PepT1 in the intestines of Eimeria infected broiler chickens. Poult. Sci. 96 (2), 465–473. 10.3382/ps/pew303 27591271

[B95] Miyashita Y. Kuraji R. Ito H. Numabe Y. (2022). Wound healing in periodontal disease induces macrophage polarization characterized by different arginine‐metabolizing enzymes. J. Periodont Res. 57 (2), 357–370. 10.1111/jre.12965 34918843

[B96] Molderings G. J. Haenisch B. (2012). Agmatine (decarboxylated L-arginine): physiological role and therapeutic potential. Pharmacol. Ther. 133 (3), 351–365. 10.1016/j.pharmthera.2011.12.005 22212617

[B97] Molecular and epigenetic basis of macrophage polarized activation. Seminars in immunology: Elsevier; 2015. 10.1016/j.smim.2015.10.003 26561250

[B98] Montanez R. Rodriguez-Caso C. Sanchez-Jimenez F. Medina M. A. (2008). *In silico* analysis of arginine catabolism as a source of nitric oxide or polyamines in endothelial cells. Amino Acids 34 (2), 223–229. 10.1007/s00726-007-0502-7 17520329

[B99] Muñoz-Fernández M. A. Fresno M. (1998). The role of tumour necrosis factor, interleukin 6, interferon-γ and inducible nitric oxide synthase in the development and pathology of the nervous system. Prog. Neurobiol. 56 (3), 307–340. 10.1016/s0301-0082(98)00045-8 9770242

[B100] Myers G. S. Rasko D. A. Cheung J. K. Ravel J. Seshadri R. DeBoy R. T. (2006). Skewed genomic variability in strains of the toxigenic bacterial pathogen, *Clostridium perfringens* . Genome Res. 16 (8), 1031–1040. 10.1101/gr.5238106 16825665 PMC1524862

[B101] Nabi F. Arain M. A. Bhutto Z. A. Shah Q. A. Bangulzai N. Ujjan N. A. (2022). Effect of early feeding of L-arginine and L-threonine on hatchability and post-hatch performance of broiler chicken. Trop. Anim. Health Prod. 54 (6), 380. 10.1007/s11250-022-03378-2 36370219

[B102] Nathan C. Xie Q. (1994). Nitric oxide synthases: roles, tolls, and controls. Cell 78 (6), 915–918. 10.1016/0092-8674(94)90266-6 7522969

[B103] National Research Council (1994a). Nutrient requirement of poultry. Washington, DC, USA: 9th revised editionNational Academic Press.

[B104] National Research Council (1994b). Nutrient requirements of poultry. National Academies Press.

[B105] Nogueira B. R. F. Sakomura N. K. Reis M. P. Leme B. B. Létourneau-Montminy M. Viana G. S. (2021). Modelling broiler requirements for lysine and arginine. Animals 11 (10), 2914. 10.3390/ani11102914 34679935 PMC8532976

[B106] Osowska S. Duchemann T. Walrand S. Paillard A. Boirie Y. Cynober L. (2006). Citrulline modulates muscle protein metabolism in old malnourished rats. Am. J. Physiology-Endocrinology Metabolism 291 (3), E582–E586. 10.1152/ajpendo.00398.2005 16608884

[B107] Phang J. M. Donald S. P. Pandhare J. Liu Y. (2008). The metabolism of proline, a stress substrate, modulates carcinogenic pathways. Amino Acids 35 (4), 681–690. 10.1007/s00726-008-0063-4 18401543

[B108] Portocarero N. Braun U. (2021). The physiological role of guanidinoacetic acid and its relationship with arginine in broiler chickens. Poult. Sci. 100 (7), 101203. 10.1016/j.psj.2021.101203 34118613 PMC8193617

[B109] Potoka D. A. Nadler E. P. Upperman J. S. Ford H. R. (2002). Role of nitric oxide and peroxynitrite in gut barrier failure. World J. Surg. 26 (7), 806–811. 10.1007/s00268-002-4056-2 11948371

[B110] Qiu Y. Yang X. Wang L. Gao K. Jiang Z. (2019). L-arginine inhibited inflammatory response and oxidative stress induced by lipopolysaccharide via arginase-1 signaling in IPEC-J2 cells. Int. J. Mol. Sci. 20 (7), 1800. 10.3390/ijms20071800 30979040 PMC6479672

[B111] Qureshi M. A. (2003). Avian macrophage and immune response: an overview. Poult. Sci. 82 (5), 691–698. 10.1093/ps/82.5.691 12762389 PMC7194945

[B112] Ravindran V. Hew L. I. Ravindran G. Bryden W. L. (1999). A comparison of ileal digesta and excreta analysis for the determination of amino acid digestibility in food ingredients for poultry. Br. Poult. Sci. 40 (2), 266–274. 10.1080/00071669987692 10465395

[B113] Ravindran V. Hew L. I. Ravindran G. Bryden W. L. (2005). Apparent ileal digestibility of amino acids in dietary ingredients for broiler chickens. Animal Sci. 81 (1), 85–97. 10.1079/asc42240085

[B114] Rochell S. J. Helmbrecht A. Parsons C. M. Dilger R. N. (2017). Interactive effects of dietary arginine and Eimeria acervulina infection on broiler growth performance and metabolism. Poult. Sci. 96 (3), 659–666. 10.3382/ps/pew295 27601684

[B115] Rodriguez P. C. Quiceno D. G. Ochoa A. C. (2007). L-arginine availability regulates T-lymphocyte cell-cycle progression. Blood 109 (4), 1568–1573. 10.1182/blood-2006-06-031856 17023580 PMC1794048

[B116] Rosenberg M. M. Zoebisch O. C. (1952). A chick test for toxicity in forage legumes 1. Agron. J. 44 (6), 315–318. 10.2134/agronj1952.00021962004400060008x

[B117] Ross-Broiler Nutrition Specifications 2022-EN (2022), Ross-Broiler Nutrition Specifications 2022-EN.

[B118] Ruan D. Fouad A. M. Fan Q. L. Huo X. H. Kuang Z. X. Wang H. (2020). Dietary L-arginine supplementation enhances growth performance, intestinal antioxidative capacity, immunity and modulates gut microbiota in yellow-feathered chickens. Poult. Sci. 99 (12), 6935–6945. 10.1016/j.psj.2020.09.042 33248609 PMC7705054

[B119] Rubin L. L. Canal C. W. Ribeiro A. Kessler A. Silva I. Trevizan L. (2007). Effects of methionine and arginine dietary levels on the immunity of broiler chickens submitted to immunological stimuli. Braz. J. Poult. Sci. 9 (4), 241–247. 10.1590/s1516-635x2007000400006

[B120] Sadeghi G. Samie A. Pourreza J. Rahmani H. R. (2004). Canavanine content and toxicity of raw and treated bitter vetch (Vicia ervilia) seeds for broiler chicken. Int. J. Poult. Sci. 3 (8), 522–529. 10.3923/ijps.2004.522.529

[B121] Sadeghi G. H. Mohammadi L. Ibrahim S. A. Gruber K. J. (2009a). Use of bitter vetch (Vicia ervilia) as a feed ingredient for poultry. Worlds Poult. Sci. J. 65 (1), 51–64. 10.1017/s004393390900004x

[B122] Sadeghi G. H. Pourreza J. Samei A. Rahmani H. (2009b). Chemical composition and some anti-nutrient content of raw and processed bitter vetch (Vicia ervilia) seed for use as feeding stuff in poultry diet. Trop. Anim. Health Prod. 41, 85–93. 10.1007/s11250-008-9159-9 19052906

[B123] Sailer M. Dahlhoff C. Giesbertz P. Eidens M. K. de Wit N. Rubio-Aliaga I. (2013). Increased plasma citrulline in mice marks diet-induced obesity and may predict the development of the metabolic syndrome. PloS one 8 (5), e63950. 10.1371/journal.pone.0063950 23691124 PMC3653803

[B124] San Martín R. Sobrevia L. (2006). Gestational diabetes and the adenosine/L-arginine/nitric oxide (ALANO) pathway in human umbilical vein endothelium. Placenta 27 (1), 1–10. 10.1016/j.placenta.2005.01.011 16310032

[B125] Satriano J. (2004). Arginine pathways and the inflammatory response: interregulation of nitric oxide and polyamines: review article. Amino Acids 26 (4), 321–329. 10.1007/s00726-004-0078-4 15290337

[B126] Schriek S. C. D. Staiger E. K. P. Michel K. P. (2007). Bioinformatic evaluation of L-arginine catabolic pathways in 24 cyanobacteria and transcriptional analysis of genes encoding enzymes of L-arginine catabolism in the cyanobacterium Synechocystis sp. PCC 6803. BMC Genomics 8, 437–465. 10.1186/1471-2164-8-437 18045455 PMC2242806

[B127] Seiler N. (1987). Functions of polyamine acetylation. Can. J. Physiol. Pharmacol. 65 (10), 2024–2035. 10.1139/y87-317 3322538

[B128] Shapouri-Moghaddam A. Mohammadian S. Vazini H. Taghadosi M. Esmaeili S. Mardani F. (2018). Macrophage plasticity, polarization, and function in health and disease. J. Cell Physiol. 233 (9), 6425–6440. 10.1002/jcp.26429 PMC13482560 29319160

[B129] Sica A. Mantovani A. (2012). Macrophage plasticity and polarization: *in vivo* veritas. J. Clin. Invest 122 (3), 787–795. 10.1172/JCI59643 22378047 PMC3287223

[B130] Sirathonpong O. Ruangpanit Y. Songserm O. Koo E. J. Attamangkune S. (2019). Determination of the optimum arginine: lysine ratio in broiler diets. Animal Prod. Sci. 59 (9), 1705–1710. 10.1071/an18049

[B131] Srinongkote S. Smriga M. Toride Y. (2004). Diet supplied with L‐lysine and L‐arginine during chronic stress of high stock density normalizes growth of broilers. Animal Sci. J. 75 (4), 339–343. 10.1111/j.1740-0929.2004.00195.x

[B132] Stuehr D. J. Santolini J. Wang Z. Wei C. Adak S. (2004). Update on mechanism and catalytic regulation in the NO synthases. J. Biol. Chem. 279 (35), 36167–36170. 10.1074/jbc.R400017200 15133020

[B133] Tamir H. Ratner S. (1963a). Enzymes of arginine metabolism in chicks. Arch. Biochem. Biophys. 102 (2), 249–258. 10.1016/0003-9861(63)90178-4 14064979

[B134] Tamir H. Ratner S. (1963b). A study of ornithine, citrulline and arginine synthesis in growing chicks. Arch. Biochem. Biophys. 102 (2), 259–269. 10.1016/0003-9861(63)90179-6 14061730

[B135] Tan B. Li X. G. Kong X. Huang R. Ruan Z. Yao K. (2009). Dietary L-arginine supplementation enhances the immune status in early-weaned piglets. Amino Acids 37, 323–331. 10.1007/s00726-008-0155-1 18712273

[B136] Tan B. Yin Y. Kong X. Li P. Li X. Gao H. (2010). L-Arginine stimulates proliferation and prevents endotoxin-induced death of intestinal cells. Amino Acids 38 (4), 1227–1235. 10.1007/s00726-009-0334-8 19669080 PMC2850530

[B137] Tan J. Applegate T. J. Liu S. Guo Y. Eicher S. D. (2014). Supplemental dietary L-arginine attenuates intestinal mucosal disruption during a coccidial vaccine challenge in broiler chickens. Br. J. Nutr. 112 (7), 1098–1109. 10.1017/S0007114514001846 25181320

[B138] Tapeh R. S. Zhandi M. Zaghari M. Akhlaghi A. (2017). Effects of guanidinoacetic acid diet supplementation on semen quality and fertility of broiler breeder roosters. Theriogenology 89, 178–182. 10.1016/j.theriogenology.2016.11.012 28043349

[B139] Torras-Llort M. Torrents D. Soriano-Garcia J. F. Gelpi J. L. Estévez R. Ferrer R. (2001). Sequential amino acid exchange across b(0,+)-like system in chicken brush border jejunum. J. Membr. Biol. 180 (3), 213–220. 10.1007/s002320010072 11337893

[B140] Tossenberger J. Rademacher M. Németh K. Halas V. Lemme A. (2016). Digestibility and metabolism of dietary guanidino acetic acid fed to broilers. Poult. Sci. 95 (9), 2058–2067. 10.3382/ps/pew083 26994189

[B141] Uyanga V. A. Jiao H. Zhao J. Wang X. Lin H. (2020). Dietary L-citrulline supplementation modulates nitric oxide synthesis and anti-oxidant status of laying hens during summer season. J. Animal Sci. Biotechnol. 11, 103–116. 10.1186/s40104-020-00507-5 PMC7549236 33062264

[B142] Uyanga V. A. Liu L. Zhao J. Wang X. Jiao H. Lin H. (2022). Central and peripheral effects of L-citrulline on thermal physiology and nitric oxide regeneration in broilers. Poult. Sci. 101 (3), 101669. 10.1016/j.psj.2021.101669 35101686 PMC8804195

[B143] Uyanga V. A. Sun L. Liu Y. Zhang M. Zhao J. Wang X. (2023). Effects of arginine replacement with L-citrulline on the arginine/nitric oxide metabolism in chickens: an animal model without urea cycle. J. Animal Sci. Biotechnol. 14 (1), 9–19. 10.1186/s40104-022-00817-w PMC9890773 36721201

[B144] van Meijl L. E. Popeijus H. E. Mensink R. P. (2010). Amino acids stimulate Akt phosphorylation, and reduce IL‐8 production and NF‐κB activity in HepG2 liver cells. Mol. Nutr. food Res. 54 (11), 1568–1573. 10.1002/mnfr.200900438 20512787

[B145] Vraneš M. Ostojić S. Tot A. Papović S. Gadžurić S. (2017). Experimental and computational study of guanidinoacetic acid self-aggregation in aqueous solution. Food Chem. 237, 53–57. 10.1016/j.foodchem.2017.05.088 28764030

[B146] Wang J. Chen L. Li P. Li X. Zhou H. Wang F. (2008). Gene expression is altered in piglet small intestine by weaning and dietary glutamine supplementation. J. Nutr. 138 (6), 1025–1032. 10.1093/jn/138.6.1025 18492829

[B147] Watford M. (2008). Glutamine metabolism and function in relation to proline synthesis and the safety of glutamine and proline supplementation. J. Nutr. 138 (10), 2003S–2007S. 10.1093/jn/138.10.2003S 18806115

[B148] Wentzel A. S. No title. Polarized innate immunity: conservation of macrophage polarization in carp 2020.

[B149] Wolfson R. L. Chantranupong L. Saxton R. A. Shen K. Scaria S. M. Cantor J. R. (2016). Sestrin2 is a leucine sensor for the mTORC1 pathway. Science 351 (6268), 43–48. 10.1126/science.aab2674 26449471 PMC4698017

[B150] Wolfson R. L. Sabatini D. M. (2017). The dawn of the age of amino acid sensors for the mTORC1 pathway. Cell metab. 26 (2), 301–309. 10.1016/j.cmet.2017.07.001 28768171 PMC5560103

[B151] Wu G. (2009). Amino acids: metabolism, functions, and nutrition. Amino Acids 37 (1), 1–17. 10.1007/s00726-009-0269-0 19301095

[B152] Wu G. (2010). Functional amino acids in growth, reproduction, and health. Adv. Nutr. 1 (1), 31–37. 10.3945/an.110.1008 22043449 PMC3042786

[B153] Wu G. Bazer F. W. Davis T. A. Kim S. W. Li P. Marc Rhoads J. (2009). Arginine metabolism and nutrition in growth, health and disease. Amino Acids 37 (1), 153–168. 10.1007/s00726-008-0210-y 19030957 PMC2677116

[B154] Wu G. Morris S. M. Jr (1998). Arginine metabolism: nitric oxide and beyond. Biochem. J. 336 (1), 1–17. 10.1042/bj3360001 9806879 PMC1219836

[B155] Zampiga M. Laghi L. Petracci M. Zhu C. Meluzzi A. Dridi S. (2018). Effect of dietary arginine to lysine ratios on productive performance, meat quality, plasma and muscle metabolomics profile in fast-growing broiler chickens. J. animal Sci. Biotechnol. 9, 79–14. 10.1186/s40104-018-0294-5 PMC6223088 30455879

[B156] Zhang B. Gan L. Shahid M. S. Lv Z. Fan H. Liu D. (2019). *In vivo* and *in vitro* protective effect of arginine against intestinal inflammatory response induced by *Clostridium perfringens* in broiler chickens. J. animal Sci. Biotechnol. 10, 73–14. 10.1186/s40104-019-0371-4 PMC6697915 31428367

[B157] Zhang B. Lv Z. Li H. Guo S. Liu D. Guo Y. (2017). Dietary l-arginine inhibits intestinal *Clostridium perfringens* colonisation and attenuates intestinal mucosal injury in broiler chickens. Br. J. Nutr. 118 (5), 321–332. 10.1017/S0007114517002094 28901890

[B158] Zhang B. Lv Z. Li Z. Wang W. Li G. Guo Y. (2018). Dietary L-arginine supplementation alleviates the intestinal injury and modulates the gut microbiota in broiler chickens challenged by *Clostridium perfringens* . Front. Microbiol. 9, 1716. 10.3389/fmicb.2018.01716 30108569 PMC6080643

[B159] Zheng P. Yu B. He J. Yu J. Mao X. Luo Y. (2017). Arginine metabolism and its protective effects on intestinal health and functions in weaned piglets under oxidative stress induced by diquat. Br. J. Nutr. 117 (11), 1495–1502. 10.1017/S0007114517001519 28701241

